# Shared alleles and genetic structures in different Thai domestic cat breeds: the possible influence of common racial origins

**DOI:** 10.1186/s44342-024-00013-4

**Published:** 2024-07-31

**Authors:** Wattanawan Jaito, Worapong Singchat, Chananya Patta, Chadaphon Thatukan, Nichakorn Kumnan, Piangjai Chalermwong, Trifan Budi, Thitipong Panthum, Wongsathit Wongloet, Pish Wattanadilokchatkun, Thanyapat Thong, Narongrit Muangmai, Kyudong Han, Prateep Duengkae, Rattanin Phatcharakullawarawat, Kornsorn Srikulnath

**Affiliations:** 1 Animal Genomics and Bioresource Research Unit (AGB Research Unit), Faculty of Science, Kasetsart University, 50 Ngamwongwan, Chatuchak, Bangkok, 10900 Thailand; 2 Sciences for Industry, Faculty of Science, Kasetsart University, 50 Ngamwongwan, Chatuchak, Bangkok, 10900 Thailand; 3 Mind Pets Animal Hospital, 169/10 Khlongsongtonnun, Latkrabang, Bangkok, 10520 Thailand; 4 Special Research Unit for Wildlife Genomics (SRUWG), Department of Forest Biology, Faculty of Forestry, Kasetsart University, 50 Ngamwongwan, Chatuchak, Bangkok, 10900 Thailand; 5 Interdisciplinary Graduate Program in Bioscience, Faculty of Science, Kasetsart University, 50 Ngamwongwan, Chatuchak, Bangkok, 10900 Thailand; 6 Department of Fishery Biology, Faculty of Fisheries, Kasetsart University, Bangkok, 10900 Thailand; 7 Department of Microbiology, Dankook University, Cheonan, 31116 Korea; 8 Bio-Medical Engineering Core Facility Research Center, Dankook University, Cheonan, 31116 Korea; 9 Laboratory of Animal Cytogenetics and Comparative Genomics (ACCG), Department of Genetics, Faculty of Science, Kasetsart University, 50 Ngamwongwan, Chatuchak, Bangkok, 10900 Thailand; 10 Center for Advanced Studies in Tropical Natural Resources, National Research University-Kasetsart University, Kasetsart University, Bangkok, 10900 Thailand

**Keywords:** Clustering, Individual identification, Siamese cat, Microsatellite

## Abstract

Over hundreds of years, cats have been domesticated and selectively bred, resulting in numerous pedigreed breeds expedited by recent cat shows and breeding associations. Concerns have been raised about the limited breeding options and the genetic implications of inbreeding, indicating challenges in maintaining genetic diversity and accurate identification in purebred cats. In this study, genetic variability and structure were examined in 5 Thai domestic cat breeds using 15 microsatellite markers and mitochondrial DNA (mtDNA) D-loop sequencing. In total, 184 samples representing the Wichien Maat (WCM), Suphalak (SL), Khao-Manee (KM), Korat (KR), and Konja (KJ) breeds were analyzed. High genetic diversity (*H*_o_ and *H*_e_ > 0.5) was observed in all breeds, and mtDNA analysis revealed two primary haplogroups (A and B) that were shared among all domestic cat breeds in Thailand and globally. However, minor differences were observed between Thai domestic cat breeds based on clustering analyses, in which a distinct genetic structure was observed in the WCM breed. This suggests that allele fixation for distinctive morphological traits has occurred in Thai domestic cat breeds that emerged in isolated regions with shared racial origins. Analysis of relationships among individuals within the breed revealed high identification efficiency in Thai domestic cat breeds (P_(ID)sibs_ < 10^−4^). Additionally, diverse and effective individual identification can be ensured by optimizing marker efficiency by using only nine loci. This comprehensive genetic characterization provides valuable insights into conservation strategies and breeding practices for Thai domestic cat breeds.

**Supplementary Information:**

The online version contains supplementary material available at 10.1186/s44342-024-00013-4.

## Introduction

Domestication of cats (*Felis catus*) has recently led to the development of pedigreed breeds through artificial selection. These breeds, which have emerged mostly within the last 150 years, vary in coat color and head shape [1–5]. Since their introduction at the first cat shows in London (1871) and New York (1881), domestic cats have proliferated globally [5, 6]. Currently, there are more than 100 recognized cat breeds worldwide, many of which are less than 75 years old, and different criteria are responsible for the variation in recognized cat breeds. Unlike other domesticated animals selected for varied traits, resulting in extreme diversity, domestic cat breeds have been bred primarily for single-gene traits that focus on aesthetic qualities, such as coat color, length, and texture [5, 7, 8]. Currently, 40–70 breeds are recognized internationally by various cat fancy organizations, with feline associations that facilitate pedigree documentation and promote legislative understanding [9–13]. The enduring popularity of domestic cats has made accurate validation of the individual and lineage essential in the breeding, sale, and purchase of purposely bred cats [9, 10]. However, the majority of domestic cats are either owned random-bred or unowned/semi-owned feral cats [14–16]. Thus, pedigree validation and cat identity confirmation are most accurately performed using DNA profiling [17–19]. Despite the increase in purebred cat populations, many breeds still have limited breeding options. A study in 2007 showed that pure breeding led to a loss of genetic diversity [17, 20]. Inbreeding, a common issue in breeding programs, increases homozygosity by descent and is used to maintain desired recessive traits. However, it may also reduce fertility, litter size, and neonatal viability [20, 21]. Inbreeding, which is particularly risky for large breeds, is exacerbated by the neutering of males before mating and the restriction of breeding to the same cattery to avoid disease [22, 23]. For over a decade, DNA testing has been used to eliminate disease-associated variants from pedigreed populations [24–27]. However, if not carefully managed, exclusive focus on this practice could significantly reduce genetic diversity and result in inbreeding depression.

In Thailand, ancient texts, such as the Cat-Book Poems (1350–1767) and the SMUD Khoi of Cats (1868–1910), identified 23 cat breeds [28–30]. However, today only five remain: Wichien Maat or Siamese cat (WCM), Suphalak (SL), Korat (KR), Khao-Manee (KM), and Konja (KJ) (Supplementary Fig. 1). WCM and KR are recognized by CFA and TICA. Such genetic distinctions have been identified in approximately 10 global cat populations, including the WCN and KR, via studies involving over thousand cats, with these differences attributed to selection and inbreeding [8, 17, 31]. Inbreeding levels in domesticated cats have been compared across European countries that share common ancestors, although they are considered separate breeds [30, 32]. Since the nineteenth century, the WCM or Siamese cat was imported to the UK. The wedge-headed type of this slender cat breed became popular by the mid-twentieth century, nearly resulting in the extinction of its original form in Europe [28]. However, the traditional WCM was re-recognized and accepted by some registries in 1995. Selective breeding of cats, often involving the mating of close relatives, increases the risk of genetic disorders and inbreeding depression. Interestingly, hereditary diseases are largely absent from the five remaining Thai domestic cat breeds, with only isolated cases being reported, indicating a distinct development process from other global breeds. The risk of hereditary disease is mitigated by planned mating between distinct relatives while taking migration, genetic drift, and founder effects into account, leading to the potential introduction of new varieties [33, 34]. Although it is possible to improve mating schemes, including mating with distinct relatives of the same breed, genetic information on domestic cat populations is often limited, especially in Thailand. To date, only a few studies have investigated microsatellite markers in Thai cats, specifically in KR and WCM breeds [32], and no studies have used mtDNA D-loop sequences. Moderate genetic diversity was revealed within both breeds through microsatellite analysis, which also suggested evidence of population structure and inbreeding [32]. However, a comprehensive understanding of genetic diversity and population structure across Thai cat breeds is still limited.

This study aimed to examine the management of five Thai domestic cat breeds from a breeder’s perspective. It is hypothesized that there are only a few Thai domestic cat breeds with a decreasing population trend, which potentially reduces the genetic diversity within each breed. If breeding issues are identified, proper planning can be implemented to maintain the genetic diversity of Thai domestic cat breeds. The present status of genetic diversity among Thai cat breeds was examined. Cluster analysis was conducted to reveal the relationships among breeds and explore regional variations in the distribution of genetic diversity. Additionally, parentage identification for breeding Thai domestic cats was established, providing a crucial parameter for maintaining or enhancing the genetic diversity within existing breeds.

## Methods

### Sample collection and DNA extraction

**Fig. 1:**
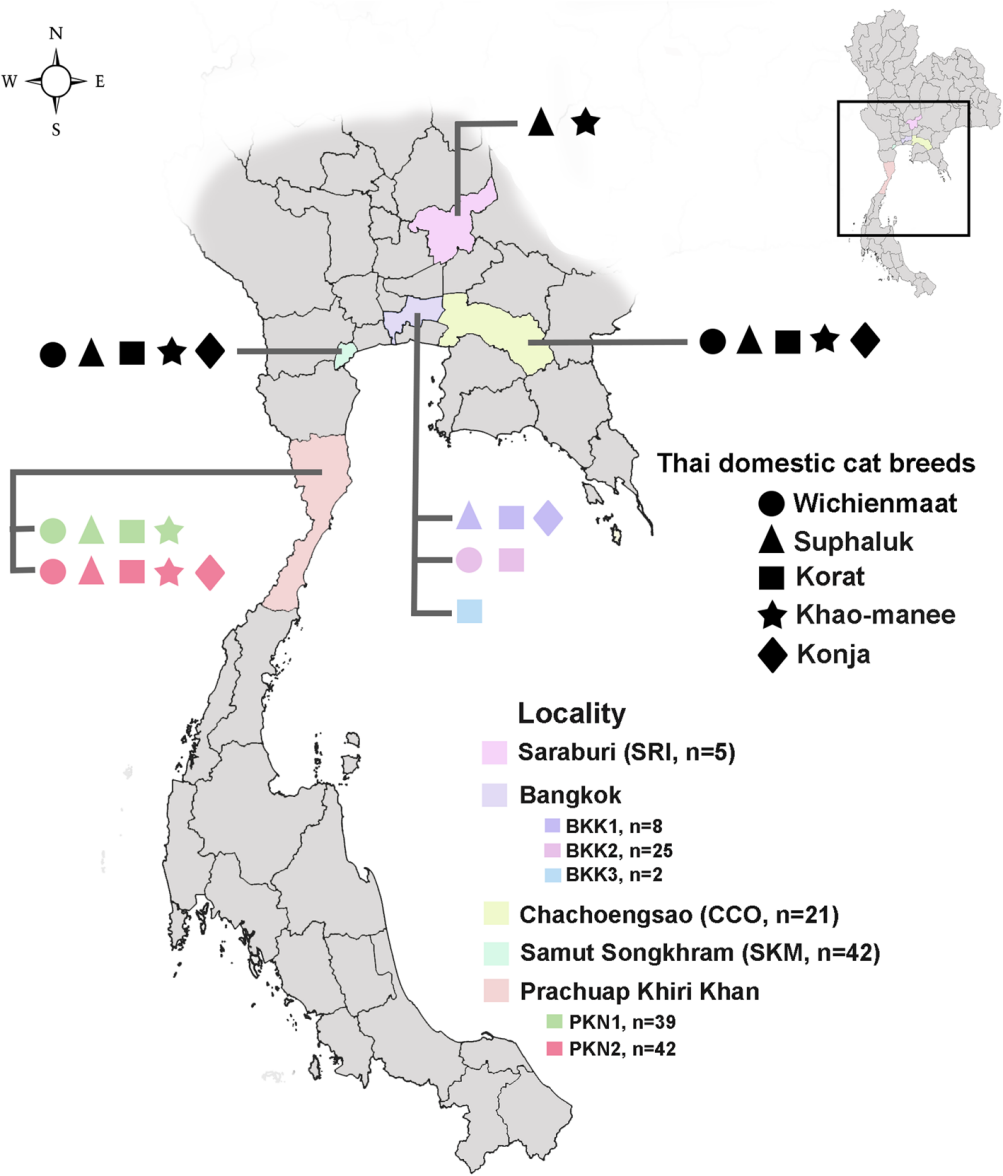
Distribution of five Thai domestic cat breeds in Thailand. Each Thai domestic cat breed is represented by a remark, while location is represented by color

A total of 184 samples, comprising 69 males and 115 females, representing 5 distinct Thai domestic cat breeds, namely, Wichien Maat (WCM), Suphalak (SL), Khao-Manee (KM), Korat (KR), and Konja (KJ), were collected from Thailand. Detailed information on the sampled individuals is presented in Fig. 1 and Supplementary Table 1. Two Scottish folds, two Persian kittens, and four domestic cats were collected as outgroups. Buccal cell samples were collected from both sides of the cheek area using a sterile swab (Thai Gauze Co., Ltd., Bangkok, Thailand) and put into dry paper Ziplock bag (Seethong 555 Co., Ltd., Samut Sakhon, Thailand) and stored at 4 °C until further use. Permission was granted by the owners of the cat farm from which the animals were obtained, and all Thai cats were promptly released after sample collection. Individuals were classified into their respective Thai domestic cat breeds by the British Library based on external morphological observations guided by Cat-Book Poems (Tamra Maew) and were then photographed (Supplementary Fig. 1) [28–30]. The picture was composed of photo images featuring 2–3 Thai cats from each farm, serving as illustrative examples of morphology (dryad: https://datadryad.org/stash/share/DWKZhrMWmgpt99NBg92Mym1YJKVs9sBpNiQ2LRqkoKI, accessed on 28 May 2024). Whole genomic DNA was isolated from buccal swab samples using a standard salting-out protocol, as described previously [35], and used as a template for polymerase chain reaction (PCR). DNA quality and quantity were assessed by 1% agarose gel electrophoresis and a NanoDrop 2000 spectrophotometer (Thermo Fisher Scientific, Wilmington, DE, USA). All experimental procedures and animal care were approved by the Animal Experiment Committee and carried out in compliance with the Regulations on Animal Experiments at Kasetsart University (approval no: ACKU65-SCI-029) and the ARRIVE guidelines (https://arriveguidelines.org).

### Microsatellite genotyping and diversity analysis

Fifteen microsatellite primer sets, originally developed for a domestic cat, were used to genotype all individuals (Supplementary Table 2) [36]. The 5′-end of the forward primer of each primer set was labeled with a fluorescent dye (carboxyfluorescein, 6-FAM, or hexachloro-fluorescein, HEX) (Macrogen Inc., Seoul, Korea). PCR amplification, product detection, and microsatellite genotyping were performed as described by Budi et al. [37] (Supplementary Table 2). Population genetics analyses were conducted using six separate datasets. These datasets included (1) 184 cats from 5 Thai cat breeds, (2) cats of the WCM breed from 5 localities, (3) cats of the SL breed from 5 localities, (4) cats of the KR breed from 7 localities, (5) cats of the KM breed from 5 localities, and (6) cats of the KJ breed from 4 localities. Population genetic parameters calculated included allelic frequency, number of alleles (*N*_a_), allelic richness (*AR*), number of effective alleles (*N*_ea_), Shannon’s information index (*I*), observed heterozygosity (*H*_o_), expected heterozygosity (*H*_e_), polymorphic information content (*PIC*), fixation index (*F*), Hardy–Weinberg equilibrium (HWE), Welch’s *t*-test (for significant differences between *H*_o_ and *H*_e_ test), linkage disequilibrium, relatedness (*r*), individual and overall inbreeding coefficients (*F*_IS_) with 95% confidence intervals (CIs), infinite allele model (IAM) based on the *F*_ST_ value with corrected *p*-values, stepwise mutation model (SMM) through *R*_ST_, the possible influence of null alleles on the estimates of genetic differentiation, *F*_ST_^ENA^ values with ENA correction for null alleles, analysis of molecular variance (AMOVA), inbreeding coefficients between breed and location (*F*_ST_), and Nei’s genetic distances between the identified groups. Analyses were performed according to Budi et al. [37]. The genotypic data from this study were deposited in the Dryad Digital Repository Dataset (https://datadryad.org/stash/share/DWKZhrMWmgpt99NBg92Mym1YJKVs9sBpNiQ2LRqkoKI, accessed on 28 May 2024).

### Genetic exchange and population structure analysis

Recent migration rates (as determined for genetic exchange) were assessed using BayesAss version 3.0.5 [38], whereas historical gene flow was calculated using MIGRATE-N 3.6.11 [39]. Wilcoxon signed-rank test was used to investigate excess heterozygosity and allelic frequency distribution shift based on two-phased model of mutation (TPM) and stepwise mutation model (SMM) using BOTTLENECK version 1.2.02 [40]. *M*-ratio test was used to explore bottlenecks from the past in the five Thai domestic cat breeds. A value < 0.68 [41] indicate a bottleneck event. For all previous runs, the evaluation and investigation of gene flow parameters followed the protocol set by Wattanadilokchatkun et al. [42]. Principal coordinate analysis (PCoA), discriminant analysis of principal components (DAPC), and STRUCTURE analysis were performed according to Budi et al. [37] using six separate datasets, as mentioned above.

### Individual identification testing

Individual identification and parentage analysis were performed on six datasets and calculated using GenAlEx version 6.5 [43] and GIMLET version 1.3.2 [44] software. The following parameters were examined.

1. Matching probability (MP) values: Probability of a random match between two unrelated individuals
  $$\text{MP }= \prod {{\text{p}}_{\text{i}}}^{2} \times \prod 2{{\text{p}}_{\text{i}}\text{p}}_{\text{j}}$$
2. Probability of identity (P_(ID)_): Probability that two randomly chosen individuals in a population have identical genotypes.

The theoretical P_(ID)_ (P_(ID)theoretical_) equation for a population in which individuals mate randomly is given as follows:

$${\text{P}}_{(\text{ID})\text{theoretical}}=2{\left(\sum {p}_{i}^{2}\right)}^{2}-\sum {p}_{i}^{4}$$

The unbiased P_(ID)_ (P_(ID)unbiased_), the less biased equation for correcting for small sample sizes, is as follows:

$${\text{P}}_{(\text{ID})\text{unbiased}}=\frac{{n}^{3}\left(2{{a}_{2}}^{2} - {a}_{4} \right) - 2{n}^{2}\left({a}_{3}+ {2a}_{2}\right) + n\left(9{a}_{2}+2\right) - 6}{(n - 1)(n - 2)(n - 3)}$$

The probability of identity between siblings (P_(ID)sibs_) is the probability that two siblings share the same multilocus genotype.

$${\text{P}}_{(\text{ID})\text{sibs}}=0.25+\left(0.5\sum {p}_{i}^{2}\right)+\left[0.5{\left(\sum {p}_{i}^{2}\right)}^{2}\right]-(0.25\sum {p}_{i}^{4})$$

(3) Probability of exclusion (PE): The probability that a potential father can be excluded as a biological father based on genetic testing results. Estimation of the probability of the genotype excluding two putative parents.
  $$\text{PE }= 1+ 4\sum {{p}_{i}}^{4}-4\sum {p}_{i}^{5}-3\sum {{p}_{i}}^{6}-8(\sum {p}_{i}^{2}{)}^{2}+8(\sum {p}_{i}^{2})(\sum {p}_{i}^{3})+2(\sum {p}_{i}^{3}{)}^{2}$$

Microsatellite markers utilized for validating individual identification were refined, emphasizing the utilization of polymorphic information content (*PIC*) values for assessing the efficacy of the two panel sets in individual identification and parentage testing. This evaluation was conducted using the ant colony optimization (ACO) algorithm proposed by Iwata and Ninomiya [45] in accordance with the methodology described by Rasoarahona et al. [46].

### Mitochondrial DNA D-loop sequencing and data analysis

The mtDNA D-loop sequences of DNA fragments were amplified using primers JHmtF3 (5′-GATAGTGCTTAATCGTGC–3′) and JHmtR3 (5′-GTCCTGTGGAACAATAGG-3′) [47]. PCR amplification, PCR product detection, and mtDNA D-loop sequencing were performed as described by Budi et al. [37] with slight modifications. The sequence datasets were separated into six datasets, as described above. Genetic diversity and demography analyses of the mtDNA d-loop region were performed as reported by Budi et al. [37]. Briefly, the process involved calculating the number of haplotypes (*H*), haplotype diversity (*h*), nucleotide diversity (*π*), the estimator theta (*S*), and average number of nucleotide differences (*k*), the genetic differentiation coefficient (*G*_ST_), Wright’s *F*-statistics for subpopulations within the total population (*F*_ST_), correlation of random haplotypes within populations (*Ф*_ST_), the average number of nucleotide substitutions per site between populations (*D*_xy_), the net nucleotide substitutions per site between populations (*D*_a_), and the median joining haplotype network reconstruction. The mismatch distribution approach, in which the observed frequency distribution of pairwise nucleotide differences among individuals is compared with expected distributions from an expanding population (small raggedness index) or a stationary population (large raggedness index), was used to test for genetic signatures of historical population expansion in the cats as previously described by Patta et al. [48]. Bayesian coalescent-based methods were also applied to evaluate historical demographic fluctuations using the extended Bayesian skyline plot (EBSP) analysis implemented using BEAST version 2.5.0 [49, 50]. The analysis applied the K80 model and a coalescent Bayesian skyline model with a prior gamma distribution. The prior date was set to 31.75 million years ago, according to fossil data of the Felidae family [51] and was analyzed as reported by Patta et al. [48]. All sequences were deposited in the DNA Data Bank of Japan (DDBJ) (https://www.ddbj.nig.ac.jp/, accessed on 08 September 2023) (accession numbers LC778518–LC778701) (Supplementary Table 1). The Thai cat haplogroup was determined by combining the mtDNA D-loop sequences from this study with the universal haplogroup of domestic cats [52]. A total of 324 mtDNA D-loop sequences (150 sequences from [52] and 184 from this study) were aligned and prepared as previously described by Budi et al. [37]. A median-joining haplotype network based on mtDNA D-loop sequences was constructed by incorporating both reference sequences and the sequences generated in this study, following the methodology described by Budi et al. [37].

A Bayesian approach implemented in MIGRATE-N v4.4.3 [53] was used to estimate migration rates and effective population sizes based on coalescent theory from mtDNA D-loop data sets and was performed as reported by Patta et al. [48]. Historical population demographic changes were determined using a statistical test of neutrality, including Tajima’s D* [54], Fu and Li’s D* and F* [55], and Fu’s *F*s [56] implemented in Arlequin version 3.5 [57].

## Results

### Genetic variability of Thai cat breed based on microsatellite data

A total of 137 alleles were observed for WCM, 110 alleles for SL, 111 alleles for KR, 95 alleles for KM, and 92 alleles for KJ, with the mean number of alleles per locus being 7.267 ± 0.222 (Table 1). The mean allelic richness value of all populations was 7.210 ± 0.241. Most allelic frequencies in the five Thai domestic cat populations did not significantly deviate from Hardy–Weinberg equilibrium or linkage disequilibrium. Although null alleles were frequently observed at 10 loci (FCA726, FCA733, FCA096, FCA229, FCA747, FCA178, FCA220, FCA596, FCA586, and FCA124), all the loci were treated similarly. The *PIC* across all breeds ranged from 0.024 to 0.814, *I*-values ranged from 0.058 to 2.066, and the five Thai cat breeds exhibited positive fixation index values (*F*) (Supplementary Table 3). The mean *H*_e_ was higher than *H*_o_ in all breeds, with *H*_o_ ranged from 0.048 to 1.000 (mean ± standard error (SE): 0.522 ± 0.025) and *H*_e_ ranged from 0.023 to 0.848 (0.692 ± 0.011) (Table 1 and Supplementary Table 3). Welch’s *t*-test revealed that *H*_o_ was significantly different from *H*_e_ (*p*-values < 0.05) as shown in Supplementary Table 4, and there were no statistical differences when comparing *H*_o_ and *H*_e_ pairs of the five breeds. A summary of the standard genetic diversity indices is provided in Table 1 and Supplementary Table 3. Considerably high genetic diversity, along with potential inbreeding, was observed in the results from five Thai domestic cats.

**Table 1:** Genetic diversity of 184 Thai domestic cats based on 15 microsatellite loci. Detailed information on all individuals is presented in Supplementary Table 1

**Breeds**		***N***	***N***_**a**_	***AR***	***N***_**e**_	***I***	***H***_**o**_	***H***_**e**_	***PIC***	***F***
WCM^a^	Mean	59	9.133	7.369	3.853	1.592	0.545	0.717	0.614	0.244
	SE	0	0.542	0.587	0.280	0.068	0.054	0.023	0.024	0.068
SL^b^	Mean	29	7.333	7.156	3.674	1.502	0.506	0.695	0.664	0.278
	SE	0	0.410	0.503	0.328	0.074	0.068	0.028	0.035	0.088
KR^c^	Mean	41	7.400	7.171	3.416	1.460	0.538	0.688	0.637	0.226
	SE	0	0.400	0.465	0.220	0.058	0.048	0.023	0.031	0.057
KM^d^	Mean	42	6.333	8.423	3.317	1.368	0.511	0.673	0.677	0.252
	SE	0	0.374	0.563	0.226	0.070	0.064	0.028	0.026	0.079
KJ^e^	Mean	37	6.133	5.933	3.493	1.429	0.511	0.690	0.641	0.268
	SE	0	0.363	0.431	0.253	0.064	0.049	0.026	0.027	0.058
All population	Mean	184	7.267	7.210	3.551	1.470	0.522	0.692	0.685	0.254
	SE	0	0.222	0.241	0.117	0.030	0.025	0.011	0.024	0.031

*N* Sample size, *N*_a_ Number of alleles, *AR* Allelic richness, *N*_e_ Number of effective alleles, *I* Shannon’s information index, *H*_o_ Observed heterozygosity, *H*_e_ Expected heterozygosity, *PIC* Polymorphic information content, *F* Fixation index

^a^*WCM* Wichien Maat cat

^b^*SL* Suphalak cat

^c^*KR* Korat cat

^d^*KM* Kao-Manee cat

^e^*KJ* Konja cat

The mean pairwise *r*-values calculated for a total of 3771 combinations of 184 individual samples from 5 Thai domestic cat breeds were − 0.015 ± 0.001 (*WCM* =  − 0.010 ± 0.001, *SL* =  − 0.021 ± 0.003, *KR* =  − 0.016 ± 0.002, *KM* =  − 0.017 ± 0.002, and KJ, − 0.032 ± 0.006). The *r*-values lay between − 0.25 and 0.25 for 3761 pairs. The remaining 10 pairs exhibited *r* > 0.25. The distribution of *r*-values for Thai cats varied among breeds, exhibiting a left skew, indicating lower pairwise *r*-values than expected under the null hypothesis of unrelated individuals by chance (Supplementary Fig. 2a). The pairwise distributions of *r* were significantly different between the WCM and KM, SL and KR, SL and KM, and KR and KJ populations. A left-skewed distribution was observed in *F*_IS_ values among the five Thai domestic cat breeds. However, the distribution of *F*_IS_ in all breeds did not differ significantly (Supplementary Fig. 2b, Supplementary Table 5). The mean *F*_IS_ was − 0.006 ± 0.076 (Table 2), with individual values of *F*_IS_ ranging from to 0.099 to 0.563. The *N*_e_ of Thai domestic cats varied among the populations (Table 2). Significant differences (*p* < 0.05) were observed in *F*_ST_ values between populations after 110 permutations (Supplementary Table 6). Nei’s genetic distances and *R*_ST_ showed that the SL cat population was closer to the other domestic cat breeds than to the WCM cat population. The AMOVA revealed 44% variation of the individuals within the population and 1% among the populations (Supplementary Table 7).

**Table 2:** Inbreeding coefficients, relatedness, effective population size and ratio of effective population size, and census population (*N*_e_*/*N) of 184 Thai domestic cat individuals (*Felis catus*) from 5 breeds

**Thai cat breeds**	**N**	***F***_**IS**_	**Relatedness (*r*)**	**Estimated *N***_**e**_	**95% CIs for *N***_**e**_	***N***_**e**_***/*N**
WCM^a^	59	−0.012	−0.011 ± 0.060	70.200	54.000–77.800	1.190
SL^b^	29	−0.014	−0.021 ± 0.066	36.900	24.700–34.600	1.272
KR^c^	41	0.014	−0.016 ± 0.058	23.700	18.900–30.400	0.578
KM^d^	37	0.0002	−0.017 ± 0.052	19.600	15.700–26.000	0.530
KJ^e^	18	−0.039	−0.032 ± 0.070	11.600	8.900–13.200	0.644

Estimates were calculated using NeEstimator version 2.1 [37], COANCESTRY version 1.0.1.9 [37], and GenAlEx version 6.5 [43]. Detailed information for all Thai domestic cat individuals is presented in Supplementary Table 1

*N* Sample size, *F*_IS_ Inbreeding coefficient, *N*_e_ Effective population size

^a^*WCM* Wichien Maat cat

^b^*SL*, Suphalak cat

^c^*KR* Korat cat

^d^*KM* Khao-Manee cat

^e^*KJ* Konja cat

**Fig. 2:**
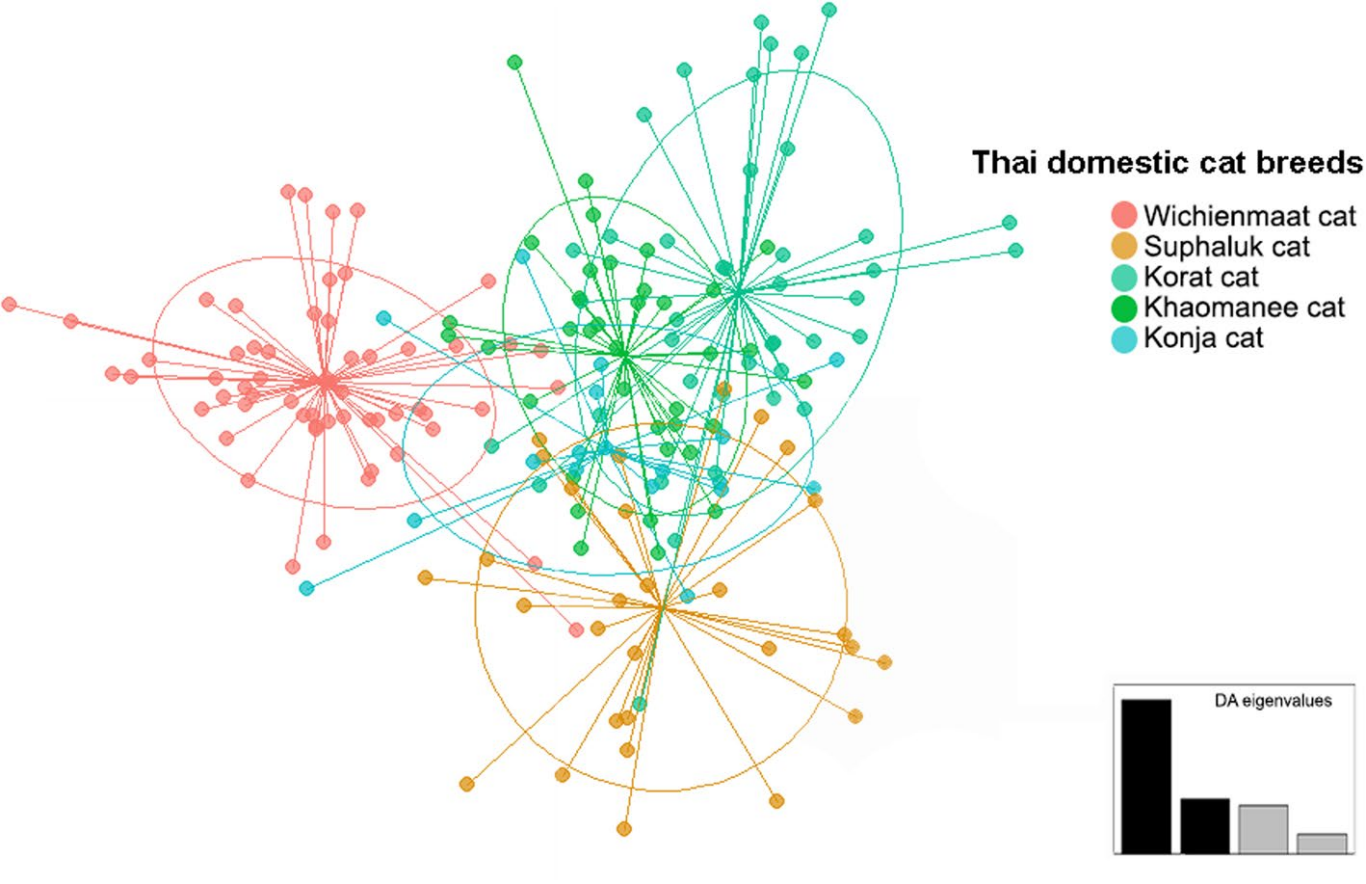
Discriminant analysis of principal components (DAPC) of five Thai domestic cat breeds (*Felis catus*). Supplementary Table 1 provides detailed information on the sampled individuals

**Fig. 3:**
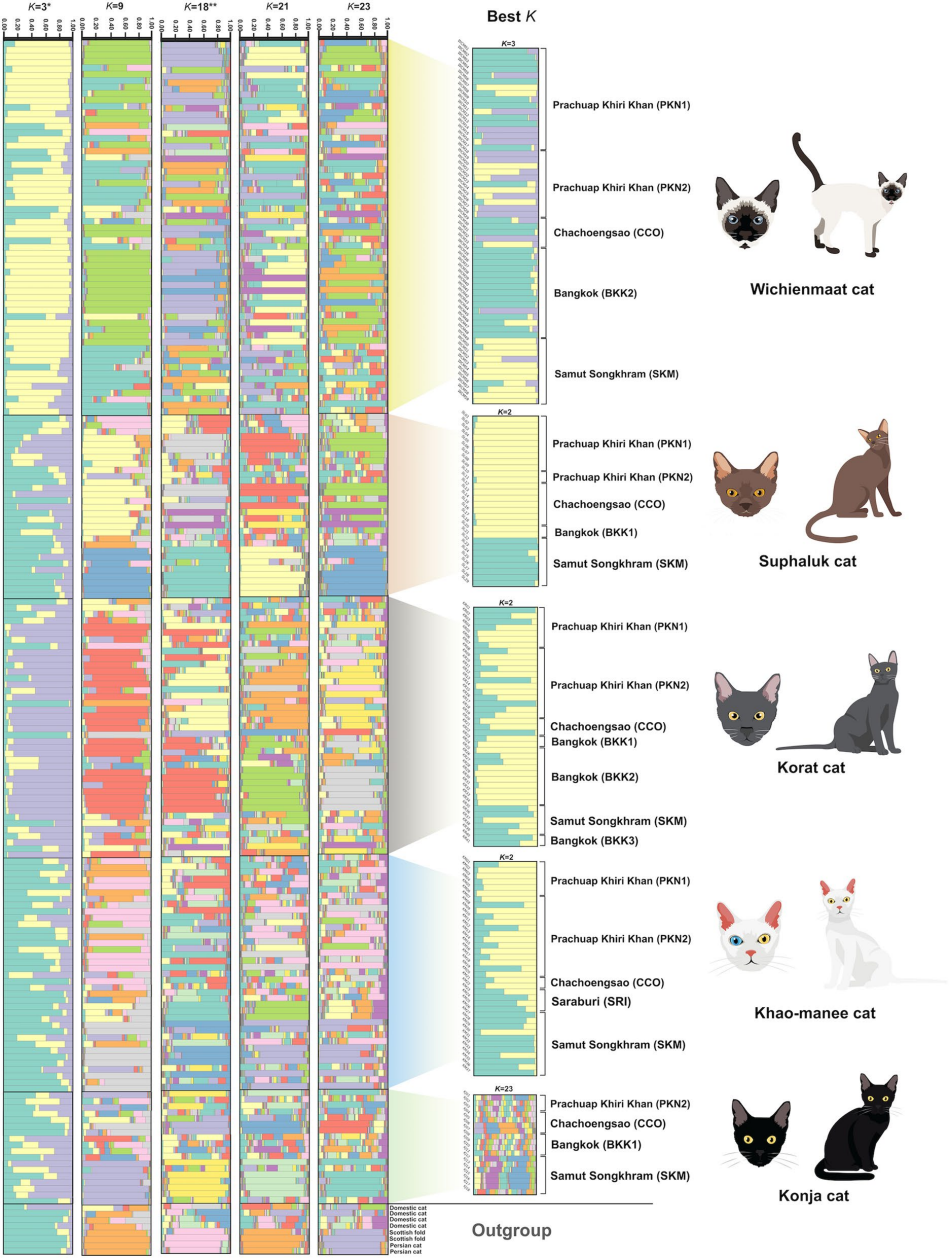
Population structure of 184 Thai domestic cat individuals (*Felis catus*). Each vertical bar on the *x*-axis represents an individual, while the *y*-axis represents the proportion of membership (posterior probability) in each genetic cluster. Thai domestic cats are superimposed on the plot, with black vertical lines indicating the boundaries. The best plot from Evanno’s Δ*K* (*) and ln P(*K*) (**). Detailed information for all Thai domestic cat individuals is presented in Supplementary Table 1

Distinct clustering patterns among the five Thai domestic cat groups were not revealed by PCoA and DAPC analysis (Fig. 2 and Supplementary Fig. 4a). These populations in PCoA analysis were differentiated using the first, second, and third principal components, which contributed 2.64%, 2.50%, and 2.13% of the total variation, respectively. Different population patterns were generated from model-based Bayesian clustering algorithms implemented in STRUCTURE with increasing *K*-values. The highest posterior probability with one peak (*K* = 3) was observed based on Evanno’s ∆*K*, while the mean ln P(*K*) revealed one peak at higher *K* value (*K* = 18) (Fig. 3). The five Thai domestic cats breeds exhibited various gene pool patterns. Two breeds, WCM and KR, showed unique gene pool patterns that differed from those of the other breeds. The standard genetic diversity indices for each Thai cat breed based on microsatellite genotyping data are presented in Supplementary Figs. 3, 4, 5, 6, 7, 8, and 9 and https://datadryad.org/stash/share/DWKZhrMWmgpt99NBg92Mym1YJKVs9sBpNiQ2LRqkoKI (accessed on 28 May 2024).

### Probability of individual identification

After analyzing all 5 Thai cat breeds, the average MP value for each locus based on the 15 microsatellite loci ranged from 1.44 × 10^−4^ to 1.00 (Table 3 and Supplementary Fig. 10a). The lowest P_(ID)_ values, including P_(ID)theoretical_, P_(ID)unbiased_, and P_(ID)sibs_, for each locus were 5.33 × 10^−2^, 5.08 × 10^−2^, and 3.51 × 10^−1^, respectively, whereas the highest values were 2.70 × 10^−1^, 2.64 × 10^−1^, and 5.69 × 10^−1^, respectively (Table 3). When considering the combination of the 15 microsatellite loci, P_(ID)sibs_ (2.20 × 10^−6^) were higher than the P_(ID)theoretical_ (3.48 × 10^−15^) and P_(ID)unbiased_ (2.09 × 10^−15^), whereas P_(ID)unbiased_ was similar to P_(ID)theoretical_ (Supplementary Fig. 11a). Furthermore, in cases where only one parent was known, locus FCA220 showed the lowest value at 45.73%, whereas locus FCA096 had the highest value at 78.56%. The panel data derived from all 15 microsatellite loci yielded a PE value of 100.00% (Supplementary Fig. 10b).

**Table 3:** Matching probability (*MP*), probability of exclusion (*PE*), theoretical probability of identity (P_(ID)theoretical_), unbiased probability of identity (P_(ID)unbiased_), and probability of identity between siblings (P_(ID)sibs_) values for each of the 15 microsatellite loci, estimated by GenAIEx version 6.5 [43] and GIMLET version 1.3.2 [44] software

**Locus**	**MP**	**PE**	**P**_**(ID)theoretical**_	**P**_**(ID)unbiased**_	**P**_**(ID)sibs**_
**FCA726**	2.52 × 10^−1^	4.84 × 10^−1^	2.35 × 10^−1^	2.31 × 10^−1^	5.24 × 10^−1^
**FCA310**	1.23 × 10^−1^	6.99 × 10^−1^	9.80 × 10^−2^	9.49 × 10^−2^	3.99 × 10^−1^
**FCA733**	1.53 × 10^−1^	6.81 × 10^−1^	1.19 × 10^−1^	1.14 × 10^−1^	4.40 × 10^−1^
**FCA096**	6.12 × 10^−2^	7.86 × 10^−1^	5.33 × 10^−2^	5.08 × 10^−2^	3.51 × 10^−1^
**FCA077**	1.15 × 10^−1^	6.91 × 10^−1^	9.22 × 10^−2^	8.95 × 10^−2^	3.89 × 10^−1^
**F42**	1.62 × 10^−1^	6.08 × 10^−1^	1.42 × 10^−1^	1.38 × 10^−1^	4.46 × 10^−1^
**FCA132**	9.37 × 10^−2^	7.18 × 10^−1^	7.65 × 10^−2^	7.37 × 10^−2^	3.77 × 10^−1^
**FCA391**	1.26 × 10^−1^	6.82 × 10^−1^	1.10 × 10^−1^	1.07 × 10^−1^	4.09 × 10^−1^
**FCA229**	1.10 × 10^−1^	6.72 × 10^−1^	1.23 × 10^−1^	1.19 × 10^−1^	4.24 × 10^−1^
**FCA747**	7.21 × 10^−2^	7.84 × 10^−1^	6.62 × 10^−2^	6.36 × 10^−2^	3.66 × 10^−1^
**FCA178**	2.26 × 10^−1^	5.52 × 10^−1^	1.84 × 10^−1^	1.80 × 10^−1^	4.85 × 10^−1^
**FCA220**	3.29 × 10^−1^	4.57 × 10^−1^	2.70 × 10^−1^	2.64 × 10^−1^	5.69 × 10^−1^
**FCA596**	1.29 × 10^−1^	7.01 × 10^−1^	9.02 × 10^−2^	8.63 × 10^−2^	4.03 × 10^−1^
**FCA586**	7.39 × 10^−2^	7.28 × 10^−1^	7.07 × 10^−2^	6.79 × 10^−2^	3.71 × 10^−1^
**FCA124**	1.20 × 10^−1^	7.42 × 10^−1^	8.33 × 10^−2^	7.94 × 10^−2^	3.98 × 10^−1^

After optimizing and reducing microsatellite markers as detailed by Rasoarahona et al. [46], the findings indicated that employing a panel comprising nine loci while maintaining an error threshold below 5% improved the efficacy of identification testing. The genotype accumulation curve indicated that the ability to distinguish distinct genotypes increased with an increase in the number of loci. Considering an error threshold of less than 5%, the ability to distinguish distinct genotypes was 98.09% with a panel set of five loci, whereas at an error threshold of less than 1%, it increased to 99.34% with a panel set of six loci (Supplementary Fig. 12a). When calculating the average MP for each locus based on the nine microsatellite loci, the value ranged from 2.87 × 10^−4^ to 1.00 (Supplementary Table 8 and Supplementary Fig. 13a). The lowest P_(ID)_ values, including P_(ID)theoretical_, P_(ID)unbiased_, and P_(ID)sibs_, for each locus were 5.94 × 10^−2^, 4.27 × 10^−2^, and 3.59 × 10^−1^, respectively, whereas the highest values were 3.07 × 10^−1^, 2.93 × 10^−1^, and 5.53 × 10^−1^, respectively (Supplementary Table 8). When considering the combination of nine microsatellite loci, all three values, namely P_(ID)sibs_ (3.20 × 10^–4^), were higher than the P_(ID)theoretical_ (1.30 × 10^–9^) and P_(ID)unbiased_ (1.65 × 10^–10^) values, whereas P_(ID)unbiased_ was similar to P_(ID)theoretical_ (Supplementary Fig. 11b). When the two putative parents were excluded, the highest PE value was 75.96% at locus FCA096, and the lowest value was 45.70% at locus FCA726 (Supplementary Table 8). When the nine microsatellite loci were combined, the PE value was 100.00% (Supplementary Fig. 13a). Additionally, the P_(ID)sibs_ curve was computed for sets of loci necessary to achieve P_(ID)sibs_ values ranging from 0.001 to 0.0001, according to a previous study by Waits et al. [58]; the microsatellite marker sets ranged from 4 (when the highest *H*_e_ value was considered) to 5 (when the lowest *H*_e_ value was considered) loci (Supplementary Fig. 14a). Further result of probability of individual identification within each of the five Thai cat breeds are presented Supplementary Figs. 10, 11, 12, 13, and 14 and in the Dryad Digital Repository Dataset (https://datadryad.org/stash/share/DWKZhrMWmgpt99NBg92Mym1YJKVs9sBpNiQ2LRqkoKI, accessed on 28 May 2024).

### Genetic variability of Thai cat populations based on mitochondrial DNA D-loop sequences

The aligned mtDNA D-loop sequence was 423 bp long. The number of haplotypes for WCM, SL, KR, KM, and KJ was six, five, eight, seven, and six, respectively. Overall haplotype and nucleotide diversities were 0.557 ± 0.068 and 0.013 ± 0.001 for WCM, 0.658 ± 0.059 and 0.016 ± 0.001 for SL, 0.778 ± 0.037 and 0.016 ± 0.001 for KR, 0.745 ± 0.052 and 0.016 ± 0.001 for KM, and 0.699 ± 0.090 and 0.015 ± 0.002 for KJ (Table 4). Considerably high mtDNA D-loop diversity was observed in the results from five Thai domestic cats. The most common haplotypes in Thai cat breeds were FC1 and FC4. Six haplotypes (FC2, FC7, FC8, FC3, FC5, and FC6) were shared among the Thai cat breeds (Supplementary Fig. 15). The haplotypes of the Thai domestic cat breeds were compared to the 12 major universal haplogroups of domestic cats (A–L) and three outliers (OL1–OL3) identified by Grahn et al. [52]. The results indicated that the WCM were most closely associated with haplogroup A (64.41%). A majority of the 28 SL samples belonged to haplogroups A (46.43%) and B (35.71%). The KR samples were mostly haplogroup A (58.54%). The 37 KM cats were identified as belonging to haplogroup A (43.24%). The KJ samples were mostly found in haplogroup A (55.55%) (Supplementary Fig. 16).

**Table 4:** Genetic diversity of mitochondrial DNA D-loop sequences in five Thai domestic cat breeds

**Breeds**	***N***	**Number of haplotypes (H)**	**Theta (per site) from *S***	**Average number of nucleotide** **Differences (*k*)**	**Overall haplotype**	**Nucleotide diversities (*π*)**
WCM^a^	59	6	0.008	5.640	0.557 ± 0.068	0.013 ± 0.001
SL^b^	29	5	0.013	6.897	0.658 ± 0.059	0.016 ± 0.001
KR^c^	41	8	0.013	6.880	0.778 ± 0.037	0.016 ± 0.001
KM^d^	37	7	0.011	6.652	0.745 ± 0.052	0.016 ± 0.001
KJ^e^	18	6	0.011	6.092	0.699 ± 0.090	0.015 ± 0.002
All populations	184	14	0.013	6.284	0.701 ± 0.031	0.015 ± 0.001

^a^*WCM* Wichien Maat or Siamese cat

^b^*SL* Suphalak cat

^c^*KR* Korat cat

^d^*KM* Khao Manee cat

^e^*KJ* Konja cat

The *F*_ST_ values between population pairs ranged from − 0.032 to 0.019, whereas the *G*_ST_ values ranged from − 0.008 to 0.056. The *Φ*_ST_ values spanned from 0.001 to 0.012, *D*_xy_ values ranged from 0.013 to 0.016, and *D*_a_ values were 0.000 for the mtDNA D-loop sequences (Supplementary Table 9). In addition, no significant differences were observed for Tajima’s D, Fu and Li’s F*, or Fu and Li’s *D* in any of the five Thai domestic cat breeds (Supplementary Table 10). Mismatch distribution analysis revealed a unimodal distribution for the five Thai domestic cat populations (Supplementary Fig. 17). The raggedness index values, which ranged from 0.086 to 0.353, were not statistically significant (Supplementary Table 10). A constant population size was determined using an mtDNA D-loop sequence-based model (Supplementary Fig. 18). Further genetic variability and differences indices for each separate Thai cat breeds were presented in Supplementary Figs. 16, 17, and 18 and https://datadryad.org/stash/share/DWKZhrMWmgpt99NBg92Mym1YJKVs9sBpNiQ2LRqkoKI (accessed on 28 May 2024).

## Discussion

Artificial selection and population dynamics make domestic cats and their breeds unique among domesticated species. Recent studies have shown that inbreeding and reliance on popular sires in closed populations lead to reduced genetic variation in many cat breeds. According to the International Maew Thai Boran Association (TIMBA) of Thailand, most Thai domestic cats are owned by unaffiliated individuals and not intended for breeding purposes. This study was conducted on 5 randomly selected domestic cat breeds and involved the typing of 15 highly polymorphic microsatellite markers. Significant deviations from the Hardy–Weinberg equilibrium were observed in the studied breeds and populations, with 13 loci showing an absence of equilibrium at *p* < 0.01, potentially due to population division and sample size. Low positive *F*-values were observed in all breeds studied, indicating the presence of multiple populations within each breed. The *H*_e_ values were significantly higher than the *H*_o_ values, indicating a trend of inbreeding. However, low *F*_IS_ and *r*-values were observed, suggesting high genetic diversity among Thai domestic cats. An average *H*_e_ of 0.692 and allelic richness of 7.210 were noted in Thai domestic cat breeds, aligning with findings related to the Havana, Norwegian Forest Cat, or British shorthair breeds (*H*_e_ values ranging from 0.55 to 0.84) but contrasting with the lower diversity often seen in Tsushima leopard cat, Sokoke cat, and Asiatic golden cat [59–62]. Haplotype network analysis of mtDNA D-loop sequences revealed that the maternal lineages of Thai domestic cats cluster with random breeds from the USA, Europe, Eastern Mediterranean, Iran/Iraq, Indian Ocean trade route, and East Asia, along with Persian, Abyssinian, and Birman cat breeds [3, 63–65]. It was found that all Thai domestic cats were classified into haplogroups A, B, C, D, K, and L, which are commonly observed in the Near Eastern wildcat, Central Asian wildcat, European wildcat, and southern African wildcat lineages [3], while 6 (WCM: 3 individuals, SL: one individual, and KJ: two individuals) of 184 individuals were unclassified. In addition, the absence of drift or isolated populations may be attributed to extensive genetic exchange between populations within the breed, facilitated by artificial migration within the Thai domestic cat community. Additionally, AMOVA results highlighted that a substantial portion of genetic variation resides within populations rather than between populations. This suggests that sufficient diversity and heterozygosity are maintained in the current population of Thai domestic cats, facilitated by breeders’ efforts to exchange genetic material. Interestingly, extensive genetic exchange and subsequent genetic mixing have led to a reduction in genetic variation across populations. However, *N*_m_ values exceeding 1.0, which imply that genetic exchange predominates over genetic drift [66], are often found in Thai domestic cat populations. Bayesian structural, PCoA, and DAPC analyses strongly suggested that genetic structuring cannot be identified in Thai domestic cats. This was consistent with the *F*_ST_ results, which indicated minimal genetic differentiation between the pairs, although significant differences were observed. WCM, the most popular breed in Thailand, is likely to cluster and reflects a specific community of owners dedicated to maintaining the breed. Can the shared gene pool patterns observed across different domestic cat breeds be explained by genetic exchange?

It has been recognized by cat registries that the five Thai domestic cat breeds are “natural” [9, 28, 59]. These breeds, which are specific population isolates, allow randomly bred cats of similar origin to be used to augment their gene pools. However, based on genetic monitoring, high levels of heterozygosity were observed in the WCM or Siamese cat breed, possibly reflecting recent developments from randomly bred populations. Moreover, the other four cat breeds have also been developed with high genetic diversity. Remarkably, Thai cat owners have never practiced crossbreeding to produce hybrid cats. However, it remains unclear why the gene pools of the five Thai domestic cat breeds are shared with those of other domestic cat breeds worldwide. Many cat breeds have various genetic backgrounds, including cats of different breeds that share the same racial origins, such as exotic cats and Himalayan cats in the USA [8, 59]. Natural breeds have emerged from specific geographical regions that have undergone some degree of isolation, leading to the fixation of alleles for distinctive morphological traits. This contrasts with the “established” or “foundation breeds,” which were developed through selective breeding of natural breeds to approximate the standards set for them [9, 60, 67]. Mixing of the gene pools in the five Thai domestic cat breeds may explain this phenomenon. These instances confounded log-likelihood calculations, complicating the empirical determination of specific breeds. Moreover, the observed correlation between neutral genetic diversity from microsatellite markers and phenotypic variation in WCM breed may have been coincidental. Therefore, it is necessary to elucidate the correlation between microsatellite marker polymorphisms and phenotypic variations in specific domestic cat breeds. Alternatively, determining genome-wide single-nucleotide polymorphisms (SNPs) may provide more insight into the five Thai domestic breed populations. It is expected that more slow-evolving SNPs and relatively quick-evolving microsatellite markers will be examined for their ability to distinguish cat breeds with different patterns, origins, and ages of ancestry [19, 26]. However, concordance was not always observed between SNPs and microsatellite markers [68, 69]. In breeds that have been isolated for long periods and have large population sizes, microsatellite marker heterozygosity may have increased through mutations, a phenomenon that is not as evident with SNPs [70–72].

Although cat breeds are not differentiated by microsatellite genotyping, parentage analysis can be conducted. High heterozygosity was predominantly observed among individual cats in the community. AMOVA revealed that the percentage of variation among populations was lower than that among individuals within populations in the WCM, SL, KR, and KM breeds. This suggests that the microsatellite panel is suitable for individual identification. In Thailand, pedigree certification, which relies on breeder credibility, influences cat prices and traceability [73, 74]. Therefore, the usefulness of DNA markers for identifying domestic Thai cats must be evaluated. Despite the popularity of SNPs, microsatellite genotyping remains widely used for paternity testing because of its cost and time efficiency [19, 48, 75]. The probability of identity (P_(ID)_) was estimated to reflect the likelihood of two randomly selected individuals with identical genotypes across multiple loci. Three variations, namely P_(ID)theoretical_, P_(ID)unbiased_ with sample size correction, and P_(ID)sibs_, were calculated. These values are useful for marker selection criteria, with P_(ID)sibs_ values between 0.001 and 0.0001 deemed sufficiently low for individual identification [58]. In this study, using 15 loci, all results aligned with the established criteria. The overall Thai domestic cat breed identification has 3.51 × 10^−1^, with PI values of approximately 10 [76]. The average PI value in this study was 2.09 × 10^−15^, which was considered sufficient for individual identification of Thai domestic cats. For parentage identification in domestic cats using microsatellite markers, PE is comparable to panels used in other species, ranging from 90.08 to 99.79% across breeds and from 99.47 to 99.87% in random-bred cat populations [17]. The microsatellite panel for Thai domestic cats exhibited a PE exceeding 100.00%. The mean *PIC* values, informative at over 0.5, matched those of the other cat breeds, supporting the utility of the panel for individual identification.

The final phase of this study was to estimate the number of Thai domestic cat microsatellite loci required to achieve a suitable P_(ID)sibs_ value for individual identification. The initial panel of 15 loci was optimized using the ACO algorithm for time efficiency, cost-effectiveness, and discrimination power while considering *PIC* [46]. The results indicated that a panel set of only nine microsatellite loci was effective for identification testing abilities, with a *PIC* of 0.689, when considering an error threshold of less than 5%. The FCA310, FCA077, F42, FCA391, FCA747, and FCA220 loci were excluded from the panel analysis. P_(ID)sibs_ values of 3.59 × 10^−1^ were observed for the 9 loci panel, which was 1.02 times greater than that of the 15 loci panel. This suggested that the nine loci panel could be used to identify Thai domestic cats. The P_(ID)_ value depends on the total number of loci and their information. Ideally, combining the most polymorphic markers would be favorable; however, this can pose challenges for allele calling owing to frequent sequence and allelic variations. These findings allow more precise allelic frequency estimates, which are crucial for evaluating matching DNA profiles in Thai domestic cat breeds. Routine identification and parentage verification in many animal species are based on well-characterized microsatellite panels that have been developed because of their highly discriminatory characteristics, leading to a high rate of parental or identification exclusion. Interest in transitioning to SNPs for parentage verification and identification in production animals has been growing [77–79] and is underway for many species. Although SNPs have been recognized as important genetic markers, polymorphic microsatellite markers generally have a higher *PIC* than a given SNP [80]. Owing to their decreased variability, SNPs offer lower resolution power, requiring more SNPs to achieve the same parentage-discriminating power as an microsatellite panel [19].

This study investigated the genetic variability of Thai domestic cat breeds using microsatellite data and mtDNA D-loop sequences and found substantial genetic diversity within each breed. Analysis using advanced methods such as PCoA, DAPC, and Bayesian clustering did not detect unique genetic structuring among Thai domestic cats, highlighting the intricate nature of Thai domestic cat breed as “natural”. Distinctive morphological traits were fixed in alleles of Thai domestic cat breeds that emerged from isolated regions with shared racial origins. Additionally, mtDNA analysis revealed high haplotype diversity in the Thai domestic cat breeds, which shared the A, B, C, D, K, and L universal haplogroups. Microsatellite markers showed efficacy for individual identification, particularly when optimized for nine loci. This study establishes an important genetic foundation for identifying and conserving Thai domestic cat populations, offering practical advantages for breeders in verifying parentage, and managing genetic diversity. Conservation strategies and breeding practices involve the mating of pairs that are selected from distinct families with minimal genetic relatedness. This strategy is intended to promote the production of offspring exhibiting high genetic diversity in subsequent generations, effectively mitigating the risks associated with inbreeding. Future research should build on these results by studying more breeds and exploring different correction methods to enhance our understanding of genetics.

### Supplementary Information

Supplementary Material 1: Supplementary Fig. 1. Phenotypic characteristics of (A) Wichienmaat (WCM), (B) Suphaluk (SL), (C) Korat (KR), (D) Khao-manee (KM), and (E) Konja (KJ) cat breeds. Supplementary Fig. 2. Observed distribution of (A) pairwise relatedness (*r*) and (B) inbreeding coefficients (*F*_IS_) for 184 Thai domestic cats (*Felis catus*) plotted against the expected distributions. Supplementary Fig. 3. Observed distribution of pairwise relatedness values and inbreeding coefficients that are plotted against the expected distributions for five Thai domestic cat breeds separated by location. (A, C, E, G, and I) Pairwise relatedness values (*r*) and (B, D, F, H, and J) inbreeding coefficients (*F*_IS_). Supplementary Fig. 4. Genetic structures of five Thai domestic cat breeds separated by breed and location revealed by (A, B, D, F, H and J) principal component analysis (PCoA) and (C, G, E, I, and K) the discriminant analysis of principal components (DAPC). Supplementary Fig. 5. Population structure of Wichienmaat cat breeds separated by location. The best plot from Evanno’s Δ*K *(*) and ln P(*K*) (**). Supplementary Fig. 6. Population structure of Suphaluk cat breeds separated by location. The best plot from Evanno’s Δ*K *(*) and ln P(*K*) (**). Supplementary Fig. 7. Population structure of Korat cat breeds separated by location. The best plot from Evanno’s Δ*K *(*) and ln P(*K*) (**). Supplementary Fig. 8. Population structure of Khao-manee cat breeds separated by location. The best plot from Evanno’s Δ*K *(*) and ln P(*K*) (**). Supplementary Fig. 9. Population structure of Konja cat breeds separated by location. The best plot from Evanno’s Δ*K *(*) and ln P(*K*) (**). Supplementary Fig. 10. (A) Matching probability (MP), and (B) probability of exclusion (PE) values of 15 microsatellite loci, estimated using GenAIEx version 6.5 [1] software. Supplementary Fig. 11. The theoretical probability of identity (P_(ID)theoretical_), unbiased probability of identity (P_(ID)unbiased_), and probability of identity between siblings (P_(ID)sibs_) based on 15 microsatellite loci (A, C, E, G, I, and K) and P_(ID)theoretical_, P_(ID)unbiased_, and P_(ID)sibs_ based on microsatellite loci after decreased (B, D, F, H, J, and L) of 184 Thai domestic cats (*Felis catus*) and each Thai domestic cat breed calculated using GenAlEx version 6.5 [1] and GIMLET version 1.3.2 [2] software. Supplementary Fig. 12. Genotype accumulation curve to simulate the effects of locus drop out on genotyping. (A) All five breeds, (B) Wichienmaat cat, (C) Suphaluk cat, (D) Korat cat, (E) Khao-manee cat, and (F) Konja cat. Supplementary Fig. 13. MP (Green line) and PE (Orange line) values of decreased microsatellite loci, estimated using GenAIEx version 6.5 [1] software (A, B, C, D, E, and F). Supplementary Fig. 14. Probability of identity between siblings (P_(ID)sibs_) value computed for sets of up to 15 loci, beginning with the marker with the lowest *H*_e_ value (pink) and ending with that with the highest *H*_e_ value (green). (A) All five breeds, (B) Wichienmaat cat, (C) Suphaluk cat, (D) Korat cat, (E) Khao-manee cat, and (F) Konja cat. Supplementary Fig. 15. Haplotype network of five Thai domestic cat breeds based on mitochondrial DNA D-loop sequences. Supplementary Fig. 16. Haplogroup pattern based on mitochondrial DNA D-loop sequences of five Thai domestic cat breeds. five locations of Wichienmaat cat (A), five locations of Suphaluk cat (B), seven locations of Korat cat (C), five locations of Khao-manee cat (D), and four locations of Konja cat (E). Supplementary Fig. 17. Mismatch distribution of the mitochondrial DNA D-loop sequences in 6 datasets of the Thai domestic cat populations: (A) all populations, (B) Wicheinmaat, (C) Suphaluk, (D) Korat, (E) Khao-manee (F) Konja datasets. The *x*-axis represents the number of pairwise differences (mismatches), and the *y*-axis represents the frequency of these differences. The distribution of frequencies of observed mismatches (pink line) is compared to those of frequencies of expected mismatches (green line). Supplementary Fig. 18. The historical demographic fluctuations of the mitochondrial DNA D‐loop sequences of Thai domestic cat breeds determined using Coalescent Bayesian Skyline analysis. The median effective breeds size is delimited by the black lines. The blue shaded area delimits the upper and lower bounds of the 95% highest posterior density interval. The *x*-axis represents time in years and the *y*-axis is displayed in logarithmic scale. Supplementary Table 1. Specimen populations of the five Thai domestic cat breeds included in this study. All sequences were deposited in the DNA Data Bank of Japan (DDBJ). Supplementary Table 2. The 15 loci of microsatellite primers sequences of Thai domestic cat. Supplementary Table 3. Genetic diversity of 184 Thai domestic cats based on 15 microsatellite loci. Detailed information on all individuals is presented in Supplementary Table 1. Supplementary Table 4. Welch’s *t*-test heterozygosity (*H*_o_) and Heterozygosity (*H*_e_) of Thai domestic cat individuals with respect to 15 microsatellite loci. Supplementary Table 5. Distributions of *r*-values and *F*_IS_ values for the five Thai domestic cat (*Felis catus*) breeds included in this study. Supplementary Table 6. Pairwise genetic differentiation (*F*_ST_), pairwise *F*_ST_^ENA^ values with ENA correction for null alleles and *R*_ST_ values using FSTAT version 2.9.3 [1] and between Thai domestic cat breeds based on 15 microsatellite loci. The number indicates *p* values, with 110 permutations. Detailed information on all Thai domestic cats are presented in Supplementary Table 1. Supplementary Table 7. Analysis of molecular variance (AMOVA) results for five Thai domestic cat breeds (*Felis catus*) based on 15 microsatellite loci using Arlequin version 3.5.2.2 [1]. Detailed information on all Thai domestic cat breeds individuals is presented in Supplementary Table 1. Supplementary Table 8. MP, PE, P_(ID)theoretical_, P_(ID)unbiased_, and P_(ID)sibs_ values for each of the decreased microsatellite loci sets, estimated using GenAIEx version 6.5 [1] and GIMLET version 1.3.2 [2] software. Supplementary Table 9. Genetic differentiation of D-loop sequences for the five That domestic cat breeds (*Felis catus*). Genetic differentiation coefficient (*G*_ST_), Wright’s F-statistics for subpopulations within the total population (*F*_ST_), *Ф*_ST_, gene flow (*N*_m_) from sequence data and haplotype data, average number of nucleotide substitutions per site between populations (*D*_xy_) and net nucleotide substitutions per site between populations (*D*_a_). Supplementary Table 10. Neutrality tests of mitochondrial D-loop sequence for Thai domestic cat (*Felis catus*).

## Data Availability

The genotypic data from this study were deposited in the Dryad Digital Repository Dataset (https://datadryad.org/stash/share/DWKZhrMWmgpt99NBg92Mym1YJKVs9sBpNiQ2LRqkoKI, accessed on 28 May 2024).
