# Supplementary material for: Shared alleles and genetic structures in different Thai domestic cat breeds: the possible influence of common racial origins
Source: Genomics Inform. 2024 Jul 31;22:12. doi: 10.1186/s44342-024-00013-4 (PMC11292921; doi:10.1186/s44342-024-00013-4)
Supplement: Supplementary file 1 — Supplementary Material 1: Supplementary Fig. 1. Phenotypic characteristics of (A) Wichienmaat (WCM), (B) Suphaluk (SL), (C) Korat (KR), (D) Khao-manee (KM), and (E) Konja (KJ) cat breeds. Supplementary Fig. 2. Observed distribution of (A) pairwise relatedness (r) and (B) inbreeding coefficients (FIS) for 184 Thai domestic cats (Felis catus) plotted against the expected distributions. Supplementary Fig. 3. Observed distribution of pairwise relatedness values and inbreeding coefficients that are plotted against the expected distributions for five Thai domestic cat breeds separated by location. (A, C, E, G, and I) Pairwise relatedness values (r) and (B, D, F, H, and J) inbreeding coefficients (FIS). Supplementary Fig. 4. Genetic structures of five Thai domestic cat breeds separated by breed and location revealed by (A, B, D, F, H and J) principal component analysis (PCoA) and (C, G, E, I, and K) the discriminant analysis of principal components (DAPC). Supplementary Fig. 5. Population structure of Wichienmaat cat breeds separated by location. The best plot from Evanno’s ΔK (*) and ln P(K) (**). Supplementary Fig. 6. Population structure of Suphaluk cat breeds separated by location. The best plot from Evanno’s ΔK (*) and ln P(K) (**). Supplementary Fig. 7. Population structure of Korat cat breeds separated by location. The best plot from Evanno’s ΔK (*) and ln P(K) (**). Supplementary Fig. 8. Population structure of Khao-manee cat breeds separated by location. The best plot from Evanno’s ΔK (*) and ln P(K) (**). Supplementary Fig. 9. Population structure of Konja cat breeds separated by location. The best plot from Evanno’s ΔK (*) and ln P(K) (**). Supplementary Fig. 10. (A) Matching probability (MP), and (B) probability of exclusion (PE) values of 15 microsatellite loci, estimated using GenAIEx version 6.5 [1] software. Supplementary Fig. 11. The theoretical probability of identity (P(ID)theoretical), unbiased probability of identity (P(ID)unbiased), and probability of [file 44342_2024_13_MOESM1_ESM.docx]

**Supplementary Table 1** Specimen collection of five Thai domestic cat breeds in this study. All sequences were deposited in the DNA Data Bank of Japan (DDBJ).

| **No.** | **Code** | **Breeds** | **Locality** | **Mitochondrial D-loop DDBJ accession number** | **Percent**  **identity** |
| --- | --- | --- | --- | --- | --- |
| 1 | TC01 | Wichienmaat (WCM) | Samroiyot, Prachuap Khiri Khan | LC778518 | 100.00 |
| 2 | TC02 | Wichienmaat (WCM) | Samroiyot, Prachuap Khiri Khan | LC778519 | 100.00 |
| 3 | TC03 | Wichienmaat (WCM) | Samroiyot, Prachuap Khiri Khan | LC778520 | 100.00 |
| 4 | TC04 | Wichienmaat (WCM) | Samroiyot, Prachuap Khiri Khan | LC778521 | 100.00 |
| 5 | TC05 | Wichienmaat (WCM) | Samroiyot, Prachuap Khiri Khan | LC778522 | 100.00 |
| 6 | TC06 | Wichienmaat (WCM) | Samroiyot, Prachuap Khiri Khan | LC778523 | 100.00 |
| 7 | TC07 | Wichienmaat (WCM) | Samroiyot, Prachuap Khiri Khan | LC778524 | 100.00 |
| 8 | TC08 | Wichienmaat (WCM) | Samroiyot, Prachuap Khiri Khan | LC778525 | 100.00 |
| 9 | TC09 | Wichienmaat (WCM) | Samroiyot, Prachuap Khiri Khan | LC778526 | 99.73 |
| 10 | TC10 | Wichienmaat (WCM) | Samroiyot, Prachuap Khiri Khan | LC778527 | 100.00 |
| 11 | TC11 | Wichienmaat (WCM) | Samroiyot, Prachuap Khiri Khan | LC778528 | 100.00 |
| 12 | TC12 | Wichienmaat (WCM) | Samroiyot, Prachuap Khiri Khan | LC778529 | 100.00 |
| 13 | TC13 | Wichienmaat (WCM) | Samroiyot, Prachuap Khiri Khan | LC778530 | 100.00 |
| 14 | TC14 | Wichienmaat (WCM) | Samroiyot, Prachuap Khiri Khan | LC778531 | 100.00 |
| 15 | TC15 | Wichienmaat (WCM) | Samroiyot, Prachuap Khiri Khan | LC778532 | 100.00 |
| 16 | TC16 | Wichienmaat (WCM) | Samroiyot, Prachuap Khiri Khan | LC778533 | 99.73 |
| 17 | TC17 | Wichienmaat (WCM) | Samroiyot, Prachuap Khiri Khan | LC778534 | 100.00 |
| 18 | TC18 | Suphaluk (SL) | Samroiyot, Prachuap Khiri Khan | LC778535 | 100.00 |
| 19 | TC19 | Suphaluk (SL) | Samroiyot, Prachuap Khiri Khan | LC778536 | 100.00 |
| 20 | TC20 | Suphaluk (SL) | Samroiyot, Prachuap Khiri Khan | LC778537 | 100.00 |
| 21 | TC21 | Suphaluk (SL) | Samroiyot, Prachuap Khiri Khan | LC778538 | 100.00 |
| 22 | TC22 | Suphaluk (SL) | Samroiyot, Prachuap Khiri Khan | LC778539 | 100.00 |
| 23 | TC23 | Suphaluk (SL) | Samroiyot, Prachuap Khiri Khan | LC778540 | 98.93 |
| 24 | TC24 | Suphaluk (SL) | Samroiyot, Prachuap Khiri Khan | LC778541 | 100.00 |
| 25 | TC25 | Suphaluk (SL) | Samroiyot, Prachuap Khiri Khan | LC778542 | 100.00 |
| 26 | TC26 | Suphaluk (SL) | Samroiyot, Prachuap Khiri Khan | LC778543 | 100.00 |
| 27 | TC27 | Khao-manee (KM) | Samroiyot, Prachuap Khiri Khan | LC778544 | 100.00 |
| 28 | TC28 | Khao-manee (KM) | Samroiyot, Prachuap Khiri Khan | LC778545 | 100.00 |
| 29 | TC29 | Khao-manee (KM) | Samroiyot, Prachuap Khiri Khan | LC778546 | 100.00 |
| 30 | TC30 | Khao-manee (KM) | Samroiyot, Prachuap Khiri Khan | LC778547 | 100.00 |
| 31 | TC31 | Khao-manee (KM) | Samroiyot, Prachuap Khiri Khan | LC778548 | 100.00 |
| 32 | TC32 | Khao-manee (KM) | Samroiyot, Prachuap Khiri Khan | LC778549 | 100.00 |
| 33 | TC33 | Korat (KR) | Samroiyot, Prachuap Khiri Khan | LC778550 | 100.00 |
| 34 | TC34 | Korat (KR) | Samroiyot, Prachuap Khiri Khan | LC778551 | 100.00 |
| 35 | TC35 | Korat (KR) | Samroiyot, Prachuap Khiri Khan | LC778552 | 100.00 |
| 36 | TC36 | Korat (KR) | Samroiyot, Prachuap Khiri Khan | LC778553 | 100.00 |
| 37 | TC37 | Korat (KR) | Samroiyot, Prachuap Khiri Khan | LC778554 | 100.00 |
| 38 | TC38 | Korat (KR) | Samroiyot, Prachuap Khiri Khan | LC778555 | 100.00 |
| 39 | TC39 | Korat (KR) | Samroiyot, Prachuap Khiri Khan | LC778556 | 100.00 |
| 40 | TC40 | Wichienmaat (WCM) | Hua Hin, Prachuap Khiri Khan | LC778557 | 100.00 |
|  |  |  |  |  |  |
| **No.** | **Code** | **Breeds** | **Locality** | **Mitochondrial D-loop DDBJ accession number** | **Percent**  **identity** |
| 41 | TC41 | Wichienmaat (WCM) | Hua Hin, Prachuap Khiri Khan | LC778558 | 100.00 |
| 42 | TC42 | Wichienmaat (WCM) | Hua Hin, Prachuap Khiri Khan | LC778559 | 100.00 |
| 43 | TC43 | Wichienmaat (WCM) | Hua Hin, Prachuap Khiri Khan | LC778560 | 100.00 |
| 44 | TC44 | Wichienmaat (WCM) | Hua Hin, Prachuap Khiri Khan | LC778561 | 100.00 |
| 45 | TC45 | Wichienmaat (WCM) | Hua Hin, Prachuap Khiri Khan | LC778562 | 100.00 |
| 46 | TC46 | Wichienmaat (WCM) | Hua Hin, Prachuap Khiri Khan | LC778563 | 100.00 |
| 47 | TC47 | Wichienmaat (WCM) | Hua Hin, Prachuap Khiri Khan | LC778564 | 100.00 |
| 48 | TC48 | Wichienmaat (WCM) | Hua Hin, Prachuap Khiri Khan | LC778565 | 100.00 |
| 49 | TC49 | Wichienmaat (WCM) | Hua Hin, Prachuap Khiri Khan | LC778566 | 100.00 |
| 50 | TC50 | Wichienmaat (WCM) | Hua Hin, Prachuap Khiri Khan | LC778567 | 100.00 |
| 51 | TC51 | Suphaluk (SL) | Hua Hin, Prachuap Khiri Khan | LC778568 | 100.00 |
| 52 | TC52 | Suphaluk (SL) | Hua Hin, Prachuap Khiri Khan | LC778569 | 100.00 |
| 53 | TC53 | Khao-manee (KM) | Hua Hin, Prachuap Khiri Khan | LC778570 | 100.00 |
| 54 | TC54 | Khao-manee (KM) | Hua Hin, Prachuap Khiri Khan | LC778571 | 100.00 |
| 55 | TC55 | Khao-manee (KM) | Hua Hin, Prachuap Khiri Khan | LC778572 | 99.73 |
| 56 | TC56 | Khao-manee (KM) | Hua Hin, Prachuap Khiri Khan | LC778573 | 100.00 |
| 57 | TC57 | Khao-manee (KM) | Hua Hin, Prachuap Khiri Khan | LC778574 | 100.00 |
| 58 | TC58 | Khao-manee (KM) | Hua Hin, Prachuap Khiri Khan | LC778575 | 100.00 |
| 59 | TC59 | Khao-manee (KM) | Hua Hin, Prachuap Khiri Khan | LC778576 | 100.00 |
| 60 | TC60 | Khao-manee (KM) | Hua Hin, Prachuap Khiri Khan | LC778577 | 100.00 |
| 61 | TC61 | Khao-manee (KM) | Hua Hin, Prachuap Khiri Khan | LC778578 | 100.00 |
| 62 | TC62 | Khao-manee (KM) | Hua Hin, Prachuap Khiri Khan | LC778579 | 100.00 |
| 63 | TC63 | Khao-manee (KM) | Hua Hin, Prachuap Khiri Khan | LC778580 | 99.73 |
| 64 | TC64 | Khao-manee (KM) | Hua Hin, Prachuap Khiri Khan | LC778581 | 100.00 |
| 65 | TC65 | Khao-manee (KM) | Hua Hin, Prachuap Khiri Khan | LC778582 | 100.00 |
| 66 | TC66 | Khao-manee (KM) | Hua Hin, Prachuap Khiri Khan | LC778583 | 100.00 |
| 67 | TC67 | Korat (KR) | Hua Hin, Prachuap Khiri Khan | LC778584 | 100.00 |
| 68 | TC68 | Korat (KR) | Hua Hin, Prachuap Khiri Khan | LC778585 | 100.00 |
| 69 | TC69 | Korat (KR) | Hua Hin, Prachuap Khiri Khan | LC778586 | 100.00 |
| 70 | TC70 | Korat (KR) | Hua Hin, Prachuap Khiri Khan | LC778587 | 100.00 |
| 71 | TC71 | Korat (KR) | Hua Hin, Prachuap Khiri Khan | LC778588 | 100.00 |
| 72 | TC72 | Korat (KR) | Hua Hin, Prachuap Khiri Khan | LC778589 | 100.00 |
| 73 | TC73 | Korat (KR) | Hua Hin, Prachuap Khiri Khan | LC778590 | 100.00 |
| 74 | TC74 | Korat (KR) | Hua Hin, Prachuap Khiri Khan | LC778591 | 100.00 |
| 75 | TC75 | Korat (KR) | Hua Hin, Prachuap Khiri Khan | LC778592 | 100.00 |
| 76 | TC76 | Korat (KR) | Hua Hin, Prachuap Khiri Khan | LC778593 | 100.00 |
| 77 | TC77 | Korat (KR) | Hua Hin, Prachuap Khiri Khan | LC778594 | 99.73 |
| 78 | TC78 | Korat (KR) | Hua Hin, Prachuap Khiri Khan | LC778595 | 100.00 |
| 79 | TC79 | Konja (KJ) | Hua Hin, Prachuap Khiri Khan | LC778596 | 100.00 |
| 80 | TC80 | Konja (KJ) | Hua Hin, Prachuap Khiri Khan | LC778597 | 99.73 |
| 81 | TC81 | Konja (KJ) | Hua Hin, Prachuap Khiri Khan | LC778598 | 100.00 |
| 82 | TC82 | Wichienmaat (WCM) | Mueang, Chachoengsao | LC778599 | 100.00 |
| 83 | TC83 | Wichienmaat (WCM) | Mueang, Chachoengsao | LC778600 | 100.00 |
| 84 | TC84 | Wichienmaat (WCM) | Mueang, Chachoengsao | LC778601 | 100.00 |
|  |  |  |  |  |  |
|  |  |  |  |  |  |
|  |  |  |  |  |  |
|  |  |  |  |  |  |
|  |  |  |  |  |  |
| **No.** | **Code** | **Breeds** | **Locality** | **Mitochondrial D-loop DDBJ accession number** | **Percent**  **identity** |
| 85 | TC85 | Wichienmaat (WCM) | Mueang, Chachoengsao | LC778602 | 99.73 |
| 86 | TC86 | Wichienmaat (WCM) | Mueang, Chachoengsao | LC778603 | 100.00 |
| 87 | TC87 | Suphaluk (SL) | Mueang, Chachoengsao | LC778604 | 100.00 |
| 88 | TC88 | Suphaluk (SL) | Mueang, Chachoengsao | LC778605 | 98.93 |
| 89 | TC89 | Suphaluk (SL) | Mueang, Chachoengsao | LC778606 | 99.73 |
| 90 | TC90 | Suphaluk (SL) | Mueang, Chachoengsao | LC778607 | 98.93 |
| 91 | TC91 | Suphaluk (SL) | Mueang, Chachoengsao | LC778608 | 100.00 |
| 92 | TC92 | Suphaluk (SL) | Mueang, Chachoengsao | LC778609 | 100.00 |
| 93 | TC93 | Suphaluk (SL) | Mueang, Chachoengsao | LC778610 | 100.00 |
| 94 | TC94 | Khao-manee (KM) | Mueang, Chachoengsao | LC778611 | 99.73 |
| 95 | TC95 | Khao-manee (KM) | Mueang, Chachoengsao | LC778612 | 99.73 |
| 96 | TC96 | Konja (KJ) | Mueang, Chachoengsao | LC778613 | 100.00 |
| 97 | TC97 | Konja (KJ) | Mueang, Chachoengsao | LC778614 | 99.73 |
| 98 | TC98 | Konja (KJ) | Mueang, Chachoengsao | LC778615 | 100.00 |
| 99 | TC99 | Konja (KJ) | Mueang, Chachoengsao | LC778616 | 100.00 |
| 100 | TC100 | Korat (KR) | Mueang, Saraburi | LC778617 | 100.00 |
| 101 | TC101 | Korat (KR) | Mueang, Saraburi | LC778618 | 99.73 |
| 102 | TC102 | Korat (KR) | Mueang, Saraburi | LC778619 | 100.00 |
| 103 | TC103 | Khao-manee (KM) | Mueang, Saraburi | LC778620 | 100.00 |
| 104 | TC104 | Khao-manee (KM) | Mueang, Saraburi | LC778621 | 100.00 |
| 105 | TC105 | Khao-manee (KM) | Mueang, Saraburi | LC778622 | 99.73 |
| 106 | TC106 | Khao-manee (KM) | Mueang, Saraburi | LC778623 | 100.00 |
| 107 | TC107 | Suphaluk | Mueang, Saraburi | LC778624 | 100.00 |
| 108 | TC108 | Korat (KR) | Bang Bon, Bangkok | LC778625 | 100.00 |
| 109 | TC109 | Korat (KR) | Bang Bon, Bangkok | LC778626 | 100.00 |
| 110 | TC110 | Suphaluk (SL) | Bang Bon, Bangkok | LC778627 | 100.00 |
| 111 | TC111 | Suphaluk (SL) | Bang Bon, Bangkok | LC778628 | 100.00 |
| 112 | TC112 | Konja (KJ) | Bang Bon, Bangkok | LC778629 | 100.00 |
| 113 | TC113 | Konja (KJ) | Bang Bon, Bangkok | LC778630 | 100.00 |
| 114 | TC114 | Konja (KJ) | Bang Bon, Bangkok | LC778631 | 100.00 |
| 115 | TC115 | Konja (KJ) | Bang Bon, Bangkok | LC778632 | 100.00 |
| 116 | TC116 | Wichienmaat (WCM) | Nong Chok, Bangkok | LC778633 | 100.00 |
| 117 | TC117 | Wichienmaat (WCM) | Nong Chok, Bangkok | LC778634 | 100.00 |
| 118 | TC118 | Wichienmaat (WCM) | Nong Chok, Bangkok | LC778635 | 100.00 |
| 119 | TC119 | Wichienmaat (WCM) | Nong Chok, Bangkok | LC778636 | 100.00 |
| 120 | TC120 | Wichienmaat (WCM) | Nong Chok, Bangkok | LC778637 | 100.00 |
| 121 | TC121 | Wichienmaat (WCM) | Nong Chok, Bangkok | LC778638 | 100.00 |
| 122 | TC122 | Wichienmaat (WCM) | Nong Chok, Bangkok | LC778639 | 100.00 |
| 123 | TC123 | Wichienmaat (WCM) | Nong Chok, Bangkok | LC778640 | 100.00 |
| 124 | TC124 | Wichienmaat (WCM) | Nong Chok, Bangkok | LC778641 | 100.00 |
| 125 | TC125 | Wichienmaat (WCM) | Nong Chok, Bangkok | LC778642 | 99.73 |
| 126 | TC126 | Wichienmaat (WCM) | Nong Chok, Bangkok | LC778643 | 100.00 |
| 127 | TC127 | Wichienmaat (WCM) | Nong Chok, Bangkok | LC778644 | 100.00 |
| 128 | TC128 | Wichienmaat (WCM) | Nong Chok, Bangkok | LC778645 | 100.00 |
|  |  |  |  |  |  |
|  |  |  |  |  |  |
|  |  |  |  |  |  |
|  |  |  |  |  |  |
| **No.** | **Code** | **Breeds** | **Locality** | **Mitochondrial D-loop DDBJ accession number** | **Percent**  **identity** |
| 129 | TC129 | Wichienmaat (WCM) | Nong Chok, Bangkok | LC778646 | 100.00 |
| 130 | TC130 | Wichienmaat (WCM) | Nong Chok, Bangkok | LC778647 | 100.00 |
| 131 | TC131 | Korat (KR) | Nong Chok, Bangkok | LC778648 | 100.00 |
| 132 | TC132 | Korat (KR) | Nong Chok, Bangkok | LC778649 | 100.00 |
| 133 | TC133 | Korat (KR) | Nong Chok, Bangkok | LC778650 | 100.00 |
| 134 | TC134 | Korat (KR) | Nong Chok, Bangkok | LC778651 | 100.00 |
| 135 | TC135 | Korat (KR) | Nong Chok, Bangkok | LC778652 | 100.00 |
| 136 | TC136 | Korat (KR) | Nong Chok, Bangkok | LC778653 | 100.00 |
| 137 | TC137 | Korat (KR) | Nong Chok, Bangkok | LC778654 | 100.00 |
| 138 | TC138 | Korat (KR) | Nong Chok, Bangkok | LC778655 | 100.00 |
| 139 | TC139 | Korat (KR) | Nong Chok, Bangkok | LC778656 | 100.00 |
| 140 | TC140 | Korat (KR) | Nong Chok, Bangkok | LC778657 | 100.00 |
| 141 | TC141 | Wichienmaat (WCM) | Amphawa, Samut Songkhram | LC778658 | 100.00 |
| 142 | TC142 | Wichienmaat (WCM) | Amphawa, Samut Songkhram | LC778659 | 100.00 |
| 143 | TC143 | Wichienmaat (WCM) | Amphawa, Samut Songkhram | LC778660 | 100.00 |
| 144 | TC144 | Wichienmaat (WCM) | Amphawa, Samut Songkhram | LC778661 | 100.00 |
| 145 | TC145 | Wichienmaat (WCM) | Amphawa, Samut Songkhram | LC778662 | 100.00 |
| 146 | TC146 | Wichienmaat (WCM) | Amphawa, Samut Songkhram | LC778663 | 100.00 |
| 147 | TC147 | Wichienmaat (WCM) | Amphawa, Samut Songkhram | LC778664 | 100.00 |
| 148 | TC148 | Wichienmaat (WCM) | Amphawa, Samut Songkhram | LC778665 | 100.00 |
| 149 | TC149 | Wichienmaat (WCM) | Amphawa, Samut Songkhram | LC778666 | 100.00 |
| 150 | TC150 | Wichienmaat (WCM) | Amphawa, Samut Songkhram | LC778667 | 100.00 |
| 151 | TC151 | Wichienmaat (WCM) | Amphawa, Samut Songkhram | LC778668 | 100.00 |
| 152 | TC152 | Khao-manee (KM) | Amphawa, Samut Songkhram | LC778669 | 100.00 |
| 153 | TC153 | Khao-manee (KM) | Amphawa, Samut Songkhram | LC778670 | 100.00 |
| 154 | TC154 | Khao-manee (KM) | Amphawa, Samut Songkhram | LC778671 | 100.00 |
| 155 | TC155 | Khao-manee (KM) | Amphawa, Samut Songkhram | LC778672 | 100.00 |
| 156 | TC156 | Khao-manee (KM) | Amphawa, Samut Songkhram | LC778673 | 100.00 |
| 157 | TC157 | Khao-manee (KM) | Amphawa, Samut Songkhram | LC778674 | 100.00 |
| 158 | TC158 | Khao-manee (KM) | Amphawa, Samut Songkhram | LC778675 | 100.00 |
| 159 | TC159 | Khao-manee (KM) | Amphawa, Samut Songkhram | LC778676 | 100.00 |
| 160 | TC160 | Khao-manee (KM) | Amphawa, Samut Songkhram | LC778677 | 100.00 |
| 161 | TC161 | Khao-manee (KM) | Amphawa, Samut Songkhram | LC778678 | 100.00 |
| 162 | TC162 | Khao-manee (KM) | Amphawa, Samut Songkhram | LC778679 | 100.00 |
| 163 | TC163 | Suphaluk (SL) | Amphawa, Samut Songkhram | LC778680 | 100.00 |
| 164 | TC164 | Suphaluk (SL) | Amphawa, Samut Songkhram | LC778681 | 100.00 |
| 165 | TC165 | Suphaluk (SL) | Amphawa, Samut Songkhram | LC778682 | 99.73 |
| 166 | TC166 | Suphaluk (SL) | Amphawa, Samut Songkhram | LC778683 | 100.00 |
| 167 | TC167 | Suphaluk (SL) | Amphawa, Samut Songkhram | LC778684 | 100.00 |
| 168 | TC168 | Suphaluk (SL) | Amphawa, Samut Songkhram | LC778685 | 100.00 |
| 169 | TC169 | Suphaluk (SL) | Amphawa, Samut Songkhram | LC778686 | 100.00 |
| 170 | TC170 | Suphaluk (SL) | Amphawa, Samut Songkhram | LC778687 | 100.00 |
| 171 | TC171 | Korat (KR) | Amphawa, Samut Songkhram | LC778688 | 100.00 |
|  |  |  |  |  |  |
|  |  |  |  |  |  |
|  |  |  |  |  |  |
|  |  |  |  |  |  |
|  |  |  |  |  |  |
|  |  |  |  |  |  |
| **No.** | **Code** | **breeds** | **Locality** | **Mitochondrial D-loop DDBJ accession number** | **Percent**  **identity** |
| 172 | TC172 | Korat (KR) | Amphawa, Samut Songkhram | LC778689 | 100.00 |
| 173 | TC173 | Korat (KR) | Amphawa, Samut Songkhram | LC778690 | 100.00 |
| 174 | TC174 | Korat (KR) | Amphawa, Samut Songkhram | LC778691 | 100.00 |
| 175 | TC175 | Korat (KR) | Amphawa, Samut Songkhram | LC778692 | 100.00 |
| 176 | TC176 | Konja (KJ) | Amphawa, Samut Songkhram | LC778693 | 100.00 |
| 177 | TC177 | Konja (KJ) | Amphawa, Samut Songkhram | LC778694 | 100.00 |
| 178 | TC178 | Konja (KJ) | Amphawa, Samut Songkhram | LC778695 | 100.00 |
| 179 | TC179 | Konja (KJ) | Amphawa, Samut Songkhram | LC778696 | 100.00 |
| 180 | TC180 | Konja (KJ) | Amphawa, Samut Songkhram | LC778697 | 100.00 |
| 181 | TC181 | Konja (KJ) | Amphawa, Samut Songkhram | LC778698 | 100.00 |
| 182 | TC182 | Konja (KJ) | Amphawa, Samut Songkhram | LC778699 | 100.00 |
| 183 | TC183 | Korat (KR) | Bang Khen, Bangkok | LC778700 | 100.00 |
| 184 | TC184 | Korat (KR) | Bang Khen, Bangkok | LC778701 | 100.00 |

**Supplementary Table 2** The 15 loci of microsatellite primers sequences of Thai domestic cat.

| **Primer** | **Fluorescence** |  | **Primer sequence 5′ to 3′** | | **Size (bp)** | **Ta (˚C)** |
| --- | --- | --- | --- | --- | --- | --- |
|  |  |  | **Reward** | **Forward** |  |  |
| **FCA726** | FAM |  | GCACAGAGGATTCCCCATAA | GCCCCTGTTTGCTGTGTACT | 229-245 | 57 |
| **FCA310** | FAM |  | TTAATTGTATCCCAAGTGGTCA | TAATGCTGCAATGTAGGGCA | 121-137 | 55 |
| **FCA733** | FAM |  | GAAGATGTGGGATAGATACACCAA | TGTTCAGAGGGCAAATTTCA | 183-223 | 62 |
| **FCA096** | HEX |  | CACGCCAAACTCTATGCTGA | CAATGTGCCGTCCAAGAAC | 184-224 | 56 |
| **FCA077** | HEX |  | GGCACCTATAACTACCAGTGTGA | ATCTCTGGGGAAATAAATTTTGG | 145-155 | 55 |
| **F42** | FAM |  | CCCACGTGGACTAATCAAAT | CACTGCACAAATTAAGAGGC | 205-231 | 55 |
| **FCA132** | FAM |  | ATCAAGGCCAACTGTCCG | GATGCCTCATTAGAAAAATGGC | 137-153 | 54 |
| **FCA391** | HEX |  | GCCTTCTAACTTCCTTGCAGA | TTTAGGTAGCCCATTTTCATCA | 237-273 | 55 |
| **FCA229** | HEX |  | CAAACTGACAAGCTTAGAGGGC | GCAGAAGTCCAATCTCAAAGTC | 160-170 | 55 |
| **FCA747** | HEX |  | GCCTCTTTGGCAACCATTAG | TCTTGGAATTACTCCTGGTAAACA | 131-159 | 55 |
| **FCA178** | FAM |  | GTGCCCCATGAATCTCACTT | TACAACTCAGGGGTCGTATGG | 257-269 | 56 |
| **FCA220** | FAM |  | CGATGGAAATTGTATCCATGG | GAATGAAGGCAGTCACAAACTG | 214-222 | 55 |
| **FCA596** | FAM |  | TTAGGAATGGATGGGCAGAG | TAAGGGGGTGGTGAATTTCA | 156-170 | 56 |
| **FCA586** | HEX |  | CATGACAAATGCTGAGAATGG | TGTATCTGGAAAGACAGAGCCT | 197-217 | 55 |
| **FCA124** | HEX |  | CCATTCCCTCCCTGTCTGTA | GCCTCAAGCCTCATTGCTAC | 110-134 | 55 |

**Supplementary Table 3** Genetic diversity of 184 Thai domestic cat individuals based on 15 microsatellite loci. Detailed information on all individuals is presented in Supplementary Table 1.

| Thai cat breeds | Locus | N | *N*_a_ | *AR* | *N*_e_ | *I* | *H*_o_ | *H*_e_ | *PIC* | *F* |
| --- | --- | --- | --- | --- | --- | --- | --- | --- | --- | --- |
| WCM ^1^ | FCA726 | 59 | 7.000 | 4.999 | 2.244 | 1.163 | 0.458 | 0.554 | 0.527 | 0.174 |
|  | FCA310 | 58 | 9.000 | 6.000 | 4.083 | 1.631 | 0.879 | 0.755 | 0.665 | -0.165 |
|  | FCA733 | 59 | 11.000 | 6.965 | 3.258 | 1.688 | 0.339 | 0.693 | 0.451 | 0.511 |
|  | FCA096 | 59 | 12.000 | 7.964 | 5.196 | 1.864 | 0.390 | 0.808 | 0.672 | 0.517 |
|  | FCA077 | 59 | 6.000 | 5.966 | 4.460 | 1.577 | 0.949 | 0.776 | 0.688 | -0.223 |
|  | F42 | 59 | 7.000 | 4.999 | 2.645 | 1.262 | 0.525 | 0.622 | 0.546 | 0.155 |
|  | FCA132 | 59 | 10.000 | 6.966 | 4.571 | 1.763 | 0.729 | 0.781 | 0.730 | 0.067 |
|  | FCA391 | 59 | 9.000 | 7.930 | 3.666 | 1.535 | 0.797 | 0.727 | 0.627 | -0.095 |
|  | FCA229 | 59 | 6.000 | 4.966 | 3.465 | 1.452 | 0.441 | 0.711 | 0.650 | 0.381 |
|  | FCA747 | 59 | 8.000 | 6.966 | 4.736 | 1.685 | 0.661 | 0.789 | 0.694 | 0.162 |
|  | FCA178 | 59 | 9.000 | 6.998 | 2.426 | 1.349 | 0.237 | 0.588 | 0.494 | 0.596 |
|  | FCA220 | 59 | 9.000 | 5.998 | 2.335 | 1.217 | 0.373 | 0.572 | 0.453 | 0.348 |
|  | FCA596 | 57 | 11.000 | 9.982 | 4.222 | 1.729 | 0.544 | 0.763 | 0.684 | 0.287 |
|  | FCA586 | 59 | 10.000 | 11.896 | 5.223 | 1.899 | 0.441 | 0.809 | 0.678 | 0.455 |
|  | FCA124 | 58 | 13.000 | 11.948 | 5.269 | 2.066 | 0.414 | 0.810 | 0.644 | 0.489 |
|  | Mean | 58.733 | 9.133 | 7.369 | 3.853 | 1.592 | 0.545 | 0.717 | 0.614 | 0.244 |
|  | SE | 0.153 | 0.542 | 0.587 | 0.280 | 0.068 | 0.054 | 0.023 | 0.024 | 0.068 |
| SL ^2^ | FCA726 | 29 | 4.000 | 5.000 | 1.940 | 0.864 | 0.517 | 0.485 | 0.766 | -0.067 |
|  | FCA310 | 29 | 8.000 | 7.930 | 3.840 | 1.613 | 1.000 | 0.740 | 0.773 | -0.352 |
|  | FCA733 | 28 | 9.000 | 9.000 | 2.835 | 1.542 | 0.321 | 0.647 | 0.708 | 0.503 |
|  | FCA096 | 29 | 7.000 | 9.897 | 6.570 | 1.913 | 0.552 | 0.848 | 0.464 | 0.349 |
|  | FCA077 | 29 | 7.000 | 5.000 | 4.226 | 1.606 | 0.966 | 0.763 | 0.723 | -0.265 |
|  | F42 | 29 | 7.000 | 4.966 | 3.705 | 1.490 | 0.448 | 0.730 | 0.715 | 0.386 |
|  | FCA132 | 29 | 8.000 | 6.999 | 4.833 | 1.741 | 0.621 | 0.793 | 0.655 | 0.217 |
|  | FCA391 | 29 | 6.000 | 8.861 | 2.988 | 1.279 | 0.621 | 0.665 | 0.752 | 0.067 |
|  | FCA229 | 29 | 8.000 | 5.965 | 3.641 | 1.596 | 0.207 | 0.725 | 0.446 | 0.715 |
|  | FCA747 | 29 | 7.000 | 7.965 | 5.289 | 1.760 | 0.793 | 0.811 | 0.349 | 0.022 |
|  | FCA178 | 29 | 6.000 | 3.965 | 2.568 | 1.274 | 0.172 | 0.611 | 0.755 | 0.718 |
|  | FCA220 | 29 | 6.000 | 6.896 | 1.911 | 1.024 | 0.241 | 0.477 | 0.804 | 0.494 |
|  | FCA596 | 29 | 8.000 | 5.999 | 2.739 | 1.432 | 0.345 | 0.635 | 0.722 | 0.457 |
|  | FCA586 | 29 | 8.000 | 9.964 | 4.426 | 1.653 | 0.241 | 0.774 | 0.766 | 0.688 |
|  | FCA124 | 29 | 11.000 | 8.931 | 3.602 | 1.740 | 0.552 | 0.722 | 0.773 | 0.236 |
|  | Mean | 28.933 | 7.333 | 7.156 | 3.674 | 1.502 | 0.506 | 0.695 | 0.664 | 0.278 |
|  | SE | 0.067 | 0.410 | 0.503 | 0.328 | 0.074 | 0.068 | 0.028 | 0.035 | 0.088 |
| KR ^3^ | FCA726 | 41 | 5.000 | 3.000 | 2.668 | 1.149 | 0.415 | 0.625 | 0.358 | 0.337 |
|  | FCA310 | 40 | 8.000 | 7.000 | 4.020 | 1.607 | 0.800 | 0.751 | 0.642 | -0.065 |
|  | FCA733 | 41 | 10.000 | 6.999 | 2.740 | 1.479 | 0.415 | 0.635 | 0.602 | 0.347 |
|  | FCA096 | 41 | 8.000 | 8.975 | 4.580 | 1.729 | 0.439 | 0.782 | 0.811 | 0.438 |
|  | FCA077 | 41 | 5.000 | 6.000 | 4.065 | 1.464 | 0.854 | 0.754 | 0.734 | -0.132 |
|  | F42 | 41 | 6.000 | 6.976 | 2.692 | 1.214 | 0.634 | 0.628 | 0.644 | -0.009 |
|  | FCA132 | 41 | 8.000 | 6.951 | 4.556 | 1.738 | 0.512 | 0.780 | 0.684 | 0.344 |
|  | FCA391 | 41 | 7.000 | 6.951 | 3.509 | 1.457 | 0.659 | 0.715 | 0.540 | 0.079 |
|  | FCA229 | 41 | 7.000 | 5.000 | 3.136 | 1.435 | 0.317 | 0.681 | 0.647 | 0.534 |
|  | FCA747 | 41 | 8.000 | 7.975 | 4.696 | 1.729 | 0.854 | 0.787 | 0.755 | -0.085 |
|  | FCA178 | 41 | 5.000 | 7.951 | 2.511 | 1.128 | 0.537 | 0.602 | 0.590 | 0.108 |
|  | FCA220 | 41 | 8.000 | 7.927 | 1.873 | 1.073 | 0.244 | 0.466 | 0.474 | 0.477 |
|  | FCA596 | 41 | 9.000 | 6.000 | 3.580 | 1.635 | 0.439 | 0.721 | 0.576 | 0.391 |
|  | FCA586 | 41 | 8.000 | 9.902 | 3.642 | 1.531 | 0.488 | 0.725 | 0.754 | 0.328 |
|  | FCA124 | 41 | 9.000 | 9.951 | 2.973 | 1.532 | 0.463 | 0.664 | 0.736 | 0.302 |
|  | Mean | 40.933 | 7.400 | 7.171 | 3.416 | 1.460 | 0.538 | 0.688 | 0.637 | 0.226 |
|  | SE | 0.067 | 0.400 | 0.465 | 0.220 | 0.058 | 0.048 | 0.023 | 0.031 | 0.057 |
|  |  |  |  |  |  |  |  |  |  |  |
| KM ^4^ | FCA726 | 35 | 4.000 | 4.000 | 2.213 | 0.955 | 0.257 | 0.548 | 0.430 | 0.531 |
|  | FCA310 | 36 | 6.000 | 7.973 | 3.713 | 1.471 | 0.889 | 0.731 | 0.714 | -0.216 |
|  | FCA733 | 37 | 9.000 | 12.891 | 3.263 | 1.554 | 0.459 | 0.694 | 0.794 | 0.338 |
|  | FCA096 | 37 | 7.000 | 8.946 | 4.381 | 1.625 | 0.432 | 0.772 | 0.814 | 0.440 |
|  | FCA077 | 37 | 6.000 | 5.000 | 3.593 | 1.407 | 0.919 | 0.722 | 0.711 | -0.273 |
|  | F42 | 37 | 6.000 | 7.919 | 3.299 | 1.343 | 0.649 | 0.697 | 0.629 | 0.069 |
|  | FCA132 | 37 | 8.000 | 8.999 | 3.940 | 1.549 | 0.622 | 0.746 | 0.728 | 0.167 |
|  | FCA391 | 37 | 6.000 | 8.892 | 4.161 | 1.583 | 0.784 | 0.760 | 0.758 | -0.032 |
|  | FCA229 | 37 | 5.000 | 6.946 | 3.263 | 1.352 | 0.189 | 0.694 | 0.586 | 0.727 |
|  | FCA747 | 37 | 7.000 | 8.945 | 4.625 | 1.686 | 0.676 | 0.784 | 0.758 | 0.138 |
|  | FCA178 | 37 | 4.000 | 8.946 | 2.124 | 0.892 | 0.351 | 0.529 | 0.656 | 0.336 |
|  | FCA220 | 37 | 5.000 | 8.971 | 1.640 | 0.822 | 0.270 | 0.390 | 0.571 | 0.307 |
|  | FCA596 | 37 | 7.000 | 10.000 | 3.115 | 1.461 | 0.649 | 0.679 | 0.734 | 0.045 |
|  | FCA586 | 37 | 7.000 | 6.973 | 3.917 | 1.530 | 0.189 | 0.745 | 0.606 | 0.746 |
|  | FCA124 | 37 | 8.000 | 10.944 | 2.507 | 1.290 | 0.324 | 0.601 | 0.668 | 0.461 |
|  | Mean | 36.800 | 6.333 | 8.423 | 3.317 | 1.368 | 0.511 | 0.673 | 0.677 | 0.252 |
|  | SE | 0.145 | 0.374 | 0.563 | 0.226 | 0.070 | 0.064 | 0.028 | 0.026 | 0.079 |
| KJ ^5^ | FCA726 | 18 | 5.000 | 4.000 | 2.356 | 1.134 | 0.389 | 0.576 | 0.507 | 0.324 |
|  | FCA310 | 18 | 4.000 | 4.000 | 3.411 | 1.284 | 0.611 | 0.707 | 0.667 | 0.135 |
|  | FCA733 | 18 | 7.000 | 8.000 | 2.817 | 1.395 | 0.500 | 0.645 | 0.569 | 0.225 |
|  | FCA096 | 18 | 6.000 | 5.000 | 4.320 | 1.590 | 0.444 | 0.769 | 0.693 | 0.422 |
|  | FCA077 | 18 | 5.000 | 6.000 | 3.256 | 1.334 | 0.833 | 0.693 | 0.691 | -0.203 |
|  | F42 | 18 | 7.000 | 6.000 | 2.919 | 1.340 | 0.556 | 0.657 | 0.547 | 0.155 |
|  | FCA132 | 18 | 6.000 | 4.000 | 2.623 | 1.254 | 0.333 | 0.619 | 0.466 | 0.461 |
|  | FCA391 | 18 | 5.000 | 5.000 | 4.563 | 1.553 | 0.611 | 0.781 | 0.694 | 0.217 |
|  | FCA229 | 18 | 6.000 | 6.000 | 3.522 | 1.438 | 0.333 | 0.716 | 0.763 | 0.534 |
|  | FCA747 | 18 | 7.000 | 6.000 | 4.836 | 1.759 | 0.778 | 0.793 | 0.659 | 0.019 |
|  | FCA178 | 18 | 5.000 | 5.000 | 3.393 | 1.386 | 0.667 | 0.705 | 0.667 | 0.055 |
|  | FCA220 | 18 | 6.000 | 6.000 | 1.733 | 0.943 | 0.222 | 0.423 | 0.464 | 0.474 |
|  | FCA596 | 18 | 7.000 | 8.000 | 3.682 | 1.581 | 0.333 | 0.728 | 0.727 | 0.542 |
|  | FCA586 | 18 | 6.000 | 6.000 | 3.522 | 1.475 | 0.333 | 0.716 | 0.708 | 0.534 |
|  | FCA124 | 18 | 10.000 | 10.000 | 5.445 | 1.963 | 0.722 | 0.816 | 0.785 | 0.115 |
|  | Mean | 18.000 | 6.133 | 5.933 | 3.493 | 1.429 | 0.511 | 0.690 | 0.641 | 0.268 |
|  | SE | 0.000 | 0.363 | 0.431 | 0.253 | 0.064 | 0.049 | 0.026 | 0.027 | 0.058 |

Sample size (N); number of alleles (*N*_a_); allelic richness (*AR*); number of effective alleles (*N*_e_); Shannon’s information index (*I*); observed heterozygosity (*H*_o_); expected heterozygosity (*H*_e_); polymorphic information content (*PIC*); fixation index (*F*). ^1^ WCM = Wichienmaas cat. ^2^ SL = Suplaluk cat. ^3^ KR = Korat cat. ^4^ KM = Khao-manee cat. ^5^ KJ = Konja cat.

**Supplementary Table 4** Welch’s *t*-test heterozygosity (*H*_o_) and Heterozygosity (*H*_e_) of Thai domestic cat individuals with respect to 15 microsatellite loci.

| **Thai cat breeds** | ***H*_o_** | ***H*_e_** | **df** | **t-test** | ***p*-value** |
| --- | --- | --- | --- | --- | --- |
| WCM ^1^ | 0.545±0.054 | 0.717±0.023 | -0.172 | -2.930 | 0.004 |
| SL^2^ | 0.506±0.068 | 0.695±0.028 | -0.189 | -2.570 | 0.014 |
| KR ^3^ | 0.538±0.048 | 0.688±0.023 | -0.150 | -2.818 | 0.007 |
| KM ^4^ | 0.511±0.049 | 0.673±0.028 | -0.162 | -2.319 | 0.025 |
| KJ ^5^ | 0.511±0.049 | 0.690±0.026 | -0.179 | -3.227 | 0.002 |

^1^ WCM = Wichienmaat cat. ^2^ SL = Suphaluk cat. ^3^ KR = Korat cat. ^4^ KM = Khao-manee cat. ^5^ KJ = Konja cat.

**Supplementary Table 5** Distributions of *r* values and *F*_IS_ values for the five Thai domestic cat (*Felis catus*) breeds included in this study.

| **Breed 1** | **Breed 2** | **Relatedness (*r*)** | | **Inbreeding coefficient (*F*_IS_)** | |
| --- | --- | --- | --- | --- | --- |
|  |  | **Density** | ***p-*value** | **Density** | ***p-*value** |
| All breeds | WCM ^1^ | 0.043 | < 0.05 | 0.049 | 0.999 |
| All breeds | SL ^2^ | 0.101 | < 0.01 | 0.121 | 0.780 |
| All breeds | KR ^3^ | 0.017 | 0.991 | 0.147 | 0.384 |
| All breeds | KM ^4^ | 0.046 | 0.177 | 0.067 | 0.994 |
| All breeds | KJ ^5^ | 0.190 | 4.706 | 0.271 | 0.141 |
| WCM | SL | 0.131 | 2.625 | 0.151 | 0.667 |
| WCM | KR | 0.052 | 0.102 | 0.178 | 0.355 |
| WCM | KM | 0.068 | < 0.05 | 0.099 | 0.952 |
| WCM | KJ | 0.216 | 4.265 | 0.292 | 0.146 |
| SL | KR | 0.104 | < 0.01 | 0.214 | 0.336 |
| SL | KM | 0.136 | < 0.01 | 0.120 | 0.927 |
| SL | KJ | 0.114 | 0.112 | 0.314 | 0.173 |
| KR | KM | 0.058 | 0.175 | 0.156 | 0.633 |
| KR | KJ | 0.179 | < 0.01 | 0.385 | < 0.05 |
| KM | KJ | 0.233 | 2.746 | 0.288 | 0.195 |

^1^ WCM = Wichienmaat cat. ^2^ SL = Suphaluk cat. ^3^ KR = Korat cat. ^4^ KM = Khao-manee cat. ^5^ KJ = Konja cat.

**Supplementary Table 6** Pairwise genetic differentiation (*F_ST_*), pairwise *F*_ST_^ENA^ values with ENA correction for null alleles and *R*_ST_ values using FSTAT version 2.9.3 [1] and between Thai domestic cat breeds based on 15 microsatellite loci. The number indicates *p* values, with 110 permutations. Detailed information on all Thai domestic cats are presented in Supplementary Table 1.

| **Combination** | ***F*_ST_** | ***F*_ST_^ENA^** | ***R*_ST_** |
| --- | --- | --- | --- |
| WCM ^1^ x SL ^2^ | 0.016^*^ | 0.009^*^ | 0.043 |
| WCM x KR ^3^ | 0.013^*^ | 0.010^*^ | 0.104 |
| WCM x KM ^4^ | 0.015^*^ | 0.018^*^ | 0.065 |
| WCM x KJ ^5^ | 0.025^*^ | 0.036^*^ | 0.031 |
| SL x KR | 0.007^*^ | 0.008^*^ | 0.126 |
| SL x KM | 0.012^*^ | 0.007^*^ | 0.078 |
| SL x KJ | 0.016^*^ | 0.031^*^ | 0.048 |
| KR x KM | 0.010^*^ | 0.006^*^ | 0.098 |
| KR x KJ | 0.021^*^ | 0.033^*^ | 0.038 |
| KM x KJ | 0.028^*^ | 0.026^*^ | 0.080 |

^*^*p* value < 0.05 ^1^ WCM = Wichienmaat cat, ^2^ SL = Suphaluk cat, ^3^ KR = Korat cat, ^4^ KM = Khao-manee cat, ^5^ KJ = Konja cat

1. Goudet JF. FSTAT (version 1.2): a computer program to calculate F-statistics. J Hered. 1995;86:485– 486.

**Supplementary Table 7** Analysis of molecular variance (AMOVA) results for five Thai domestic cat breeds (*Felis catus*) based on 15 microsatellite loci using Arlequin version 3.5.2.2 [1]. Detailed information on all Thai domestic cat breeds individuals is presented in Supplementary Table 1.

| **Source of variation** | **df** | **Sum of squares** | **Variance components** | **Percentage of variation** |
| --- | --- | --- | --- | --- |
| Among breed | 4 | 58.675 | 0.063 | 1 |
| Among individual | 179 | 1824.352 | 3.126 | 44 |
| Within individual | 184 | 725.000 | 3.940 | 55 |
| Total | 367 | 2608.027 | 7.129 | 100 |

1. Excoffier L, Lischer HE. Arlequin suite ver 3.5: a new series of programs to perform population genetics analyses under Linux and Windows. Mol Ecol Resour. 2010;10:564–567.

**Supplementary Table 8** MP, PE, P_(ID)theoretical_, P_(ID)unbiased_, and P_(ID)sibs_ values for each of the decreased microsatellite loci set, estimated by GenAIEx version 6.5 [1] and GIMLET version 1.3.2 [2] software.

| Locus | MP | PE | P_(ID)theoretical_ | P_(ID)unbiased_ | P_(ID)sibs_ |
| --- | --- | --- | --- | --- | --- |
| **FCA726** | 2.86x10^-1^ | 4.57x10^-1^ | 5.94x10^-2^ | 4.27x10^-2^ | 3.59x10^-1^ |
| **FCA733** | 1.87x10^-1^ | 6.64x10^-1^ | 6.79x10^-2^ | 4.83x10^-2^ | 3.73x10^-1^ |
| **FCA096** | 7.46x10^-2^ | 7.60x10^-1^ | 7.13x10^-2^ | 5.25x10^-2^ | 3.74x10^-1^ |
| **FCA132** | 1.10x10^-1^ | 6.75x10^-1^ | 7.79x10^-2^ | 6.14x10^-2^ | 3.76x10^-1^ |
| **FCA229** | 1.17x10^-1^ | 6.53x10^-1^ | 9.16x10^-2^ | 7.09x10^-2^ | 3.95x10^-1^ |
| **FCA178** | 2.23x10^-1^ | 5.58x10^-1^ | 9.25x10^-2^ | 7.25x10^-2^ | 3.95x10^-1^ |
| **FCA596** | 1.30x10^-1^ | 7.28x10^-1^ | 1.40x10^-1^ | 1.17x10^-1^ | 4.39x10^-1^ |
| **FCA586** | 1.02x10^-1^ | 7.05x10^-1^ | 1.59x10^-1^ | 1.40x10^-1^ | 4.49x10^-1^ |
| **FCA124** | 1.18x10^-1^ | 7.53x10^-1^ | 3.07x10^-1^ | 2.93x10^-1^ | 5.53x10^-1^ |

1. Peakall R, Smouse PE. GenAlEx 6.5: genetic analysis in Excel. Population genetic software for teaching and research–an update. Bioinformatics. 2012;28:2537–2539.

2. Valière, N. gimlet: a computer program for analysing genetic individual identification data. Mol Ecol Notes. 2002;2:377–379.

**Supplementary Table 9** Genetic differentiation of D-loop sequences for the five That domestic cat breeds (*Felis catus*). Genetic differentiation coefficient (*G*_ST_), Wright’s F-statistics for subpopulations within the total population (*F*_ST_), *Ф*_ST_, gene flow (*N*_m_) from sequence data and haplotype data, average number of nucleotide substitutions per site between populations (*D*_xy_) and net nucleotide substitutions per site between populations (*D*_a_).

| **Breed 1** | **Breed 2** | ***G*_ST_** | ***Ф*_ST_** | ***F*_ST_** | ***D*_xy_** | ***D*_a_** | ***N*_m_** |
| --- | --- | --- | --- | --- | --- | --- | --- |
| WCM | SL | 0.023 | 0.011 | 0.003^ns^ | 0.015 | 0.000 | 196.381 |
| WCM | KR | 0.047 | 0.010 | 0.019^ns^ | 0.015 | 0.000 | 26.074 |
| WCM | KM | 0.033 | 0.009 | 0.006^ns^ | 0.015 | 0.000 | 78.398 |
| WCM | KJ | 0.001 | 0.001 | -0.032^ns^ | 0.013 | 0.000 | Infinite |
| SL | KR | 0.056 | 0.010 | -0.008^ns^ | 0.016 | 0.000 | Infinite |
| SL | KM | 0.052 | 0.011 | -0.012^ns^ | 0.016 | 0.000 | Infinite |
| SL | KJ | -0.008 | 0.008 | -0.030^ns^ | 0.015 | 0.000 | Infinite |
| KR | KM | 0.030 | 0.012 | -0.003^ns^ | 0.016 | 0.000 | Infinite |
| KR | KJ | 0.036 | 0.009 | -0.012^ns^ | 0.015 | 0.000 | Infinite |
| KM | KJ | 0.031 | 0.006 | -0.027^ns^ | 0.015 | 0.000 | Infinite |

* *p* < 0.01. ^1^ WCM = Wichien maat or Siamese cat ^2^ SL = Suphaluk cat ^3^ KR = Korat cat ^4^ KM = Khao Manee cat ^5^ KJ = Konja cat

**Supplementary Table 10** Neutrality tests of mitochondrial D-loop sequence for Thai domestic cat (*Felis catus*).

| **Breeds** | **Tajima** | | **Fu *D**** | **Fu *F**** | **Fu's *F_s_*** | **Ewens-Watterson test** | **Chakraborty’s test** | **Ramos-Onsins and Rozas** | **Raggedness index** |
| --- | --- | --- | --- | --- | --- | --- | --- | --- | --- |
| WCM^1^ | | 1.923^ns^ | 0.689^ns^ | 1.326^ns^ | 9.156^ns^ | 0.748^ns^ | 0.260 | 0.175 | 0.215 |
| SL^2^ | | 1.021^ns^ | 0.159^ns^ | 0.508^ns^ | 8.875^ns^ | 0.501^ns^ | 0.584 | 0.160 | 0.353 |
| KR^3^ | | 0.936^ns^ | 0.469^ns^ | 0.743^ns^ | 7.358^ns^ | 0.335^ns^ | 0.684 | 0.146 | 0.086 |
| KM^4^ | | 1.538^ns^ | 0.237^ns^ | 0.786^ns^ | 6.539^ns^ | 0.458^ns^ | 0.571 | 0.173 | 0.190 |
| KJ^5^ | | 1.182^ns^ | -0.023^ns^ | 0.372^ns^ | 5.273^ns^ | 0.759^ns^ | 0.377 | 0.177 | 0.158 |
| All breeds | | 0.607^ns^ | -0.688^ns^ | 0.747^ns^ | 7.440^ns^ | 0.560^ns^ | 0.495 | 0.104 | 0.090 |

ns = not significant ^1^ WCM = Wichien maat or Siamese cat ^2^SL = Suphaluk cat ^3^KR= Korat cat ^4^KM= Khao Manee cat ^5^KJ= Konja cat


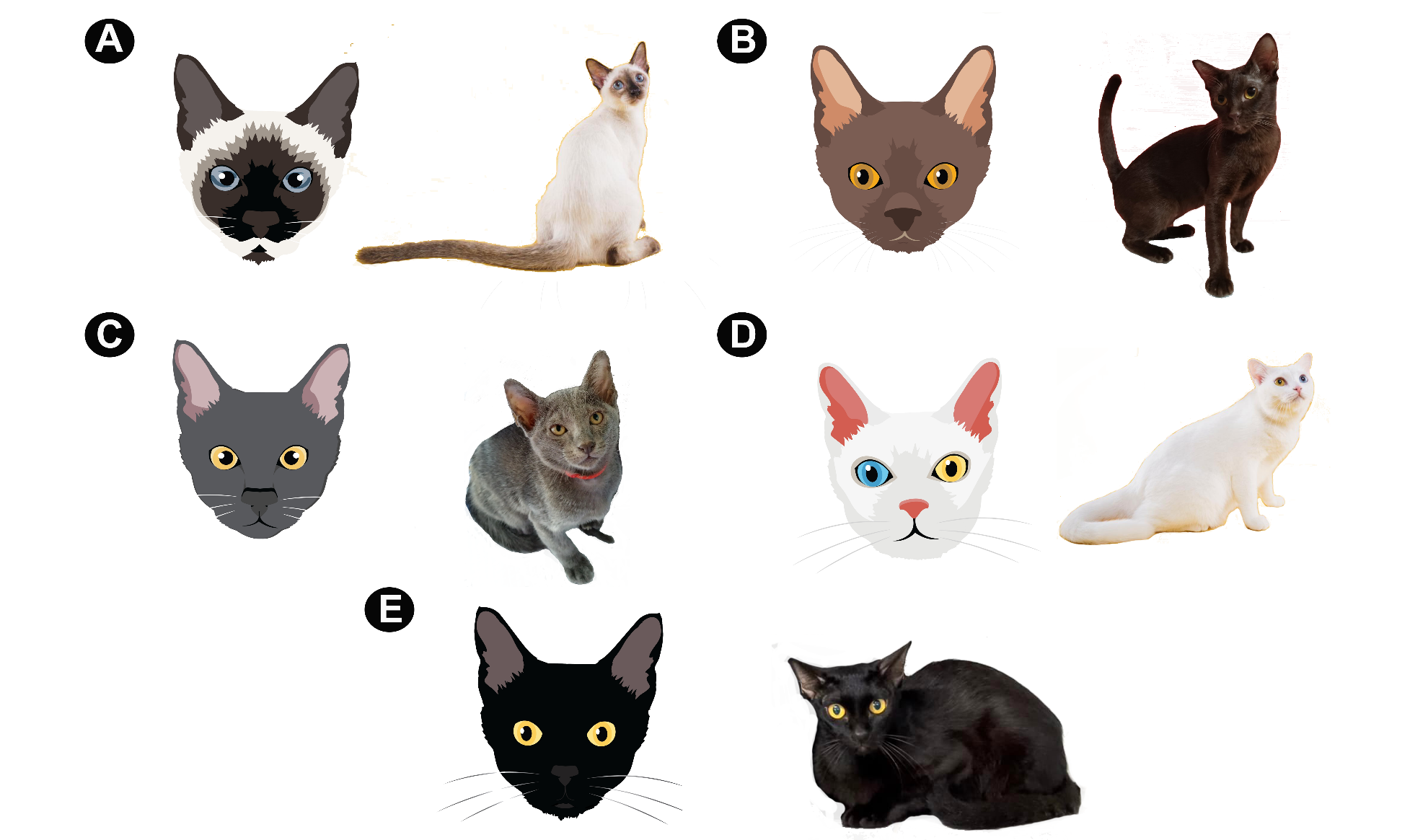


**Supplementary Fig. 1** Phenotypic characteristics of (A) Wichienmaat (WCM), (B) Suphaluk (SL), (C) Korat (KR), (D) Khao-manee (KM), and (E) Konja (KJ) cat breeds.


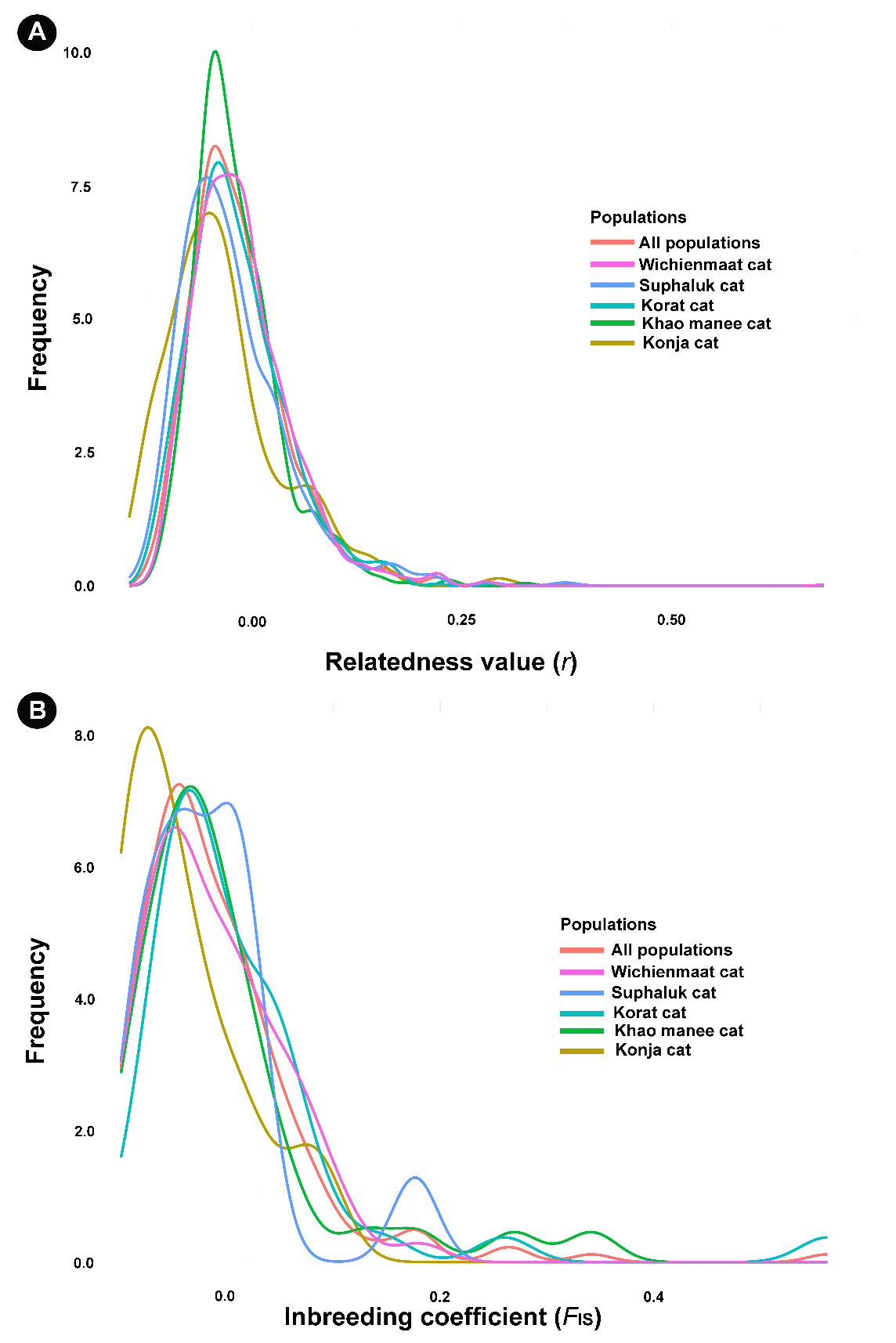


**Supplementary Fig.** **2** Observed distribution of (A) pairwise relatedness (*r*) and (B) inbreeding coefficients (*F*_IS_) for 184 Thai domestic cats (*Felis catus*) plotted against the expected distributions.


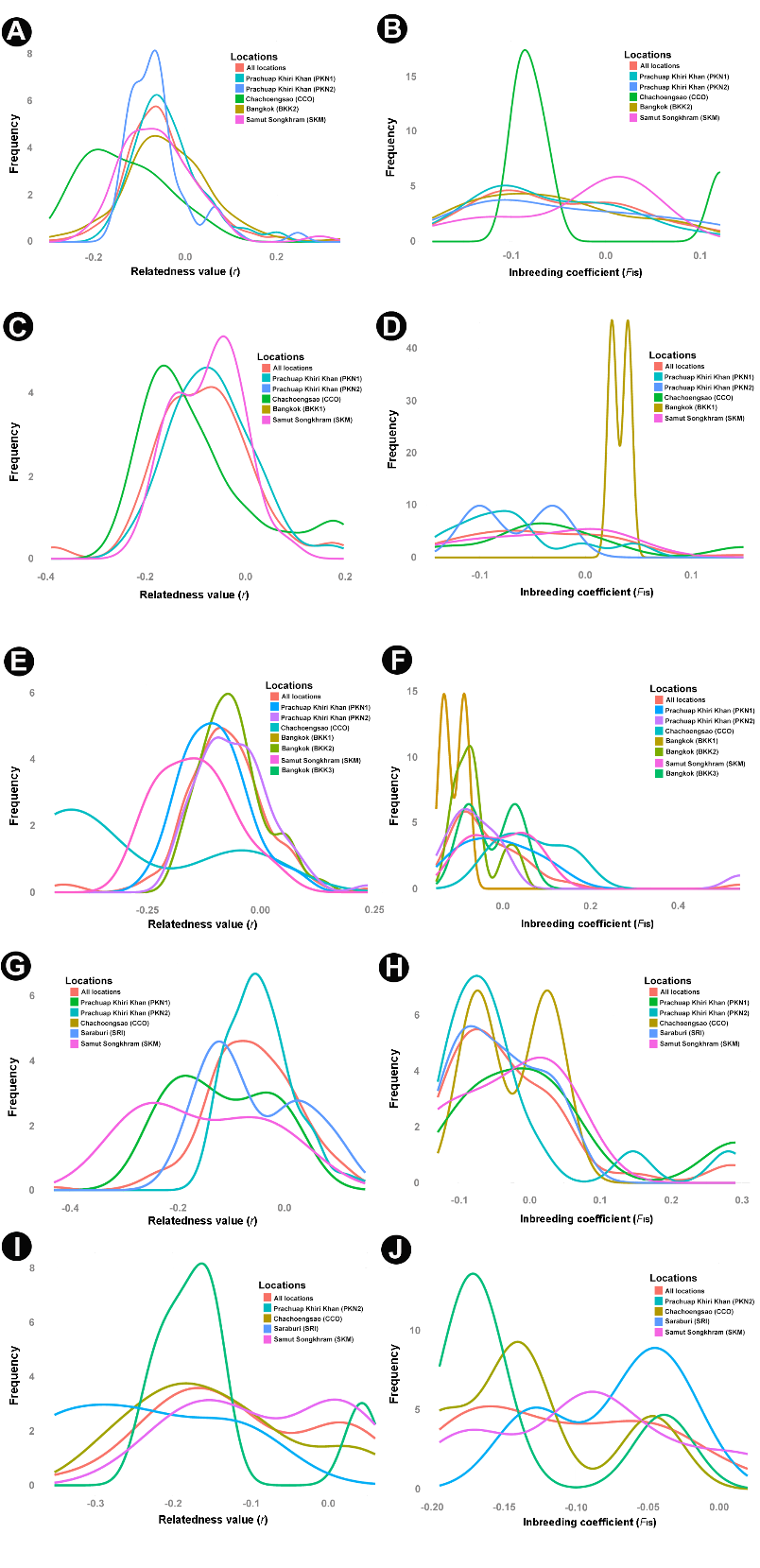


**Supplementary Fig. 3** Observed distribution of pairwise relatedness values and inbreeding coefficients that are plotted against the expected distributions for five Thai domestic cat breeds separated by location. (A, C, E, G, and I) Pairwise relatedness values (*r*) and (B, D, F, H, and J) inbreeding coefficients (*F*_IS_).


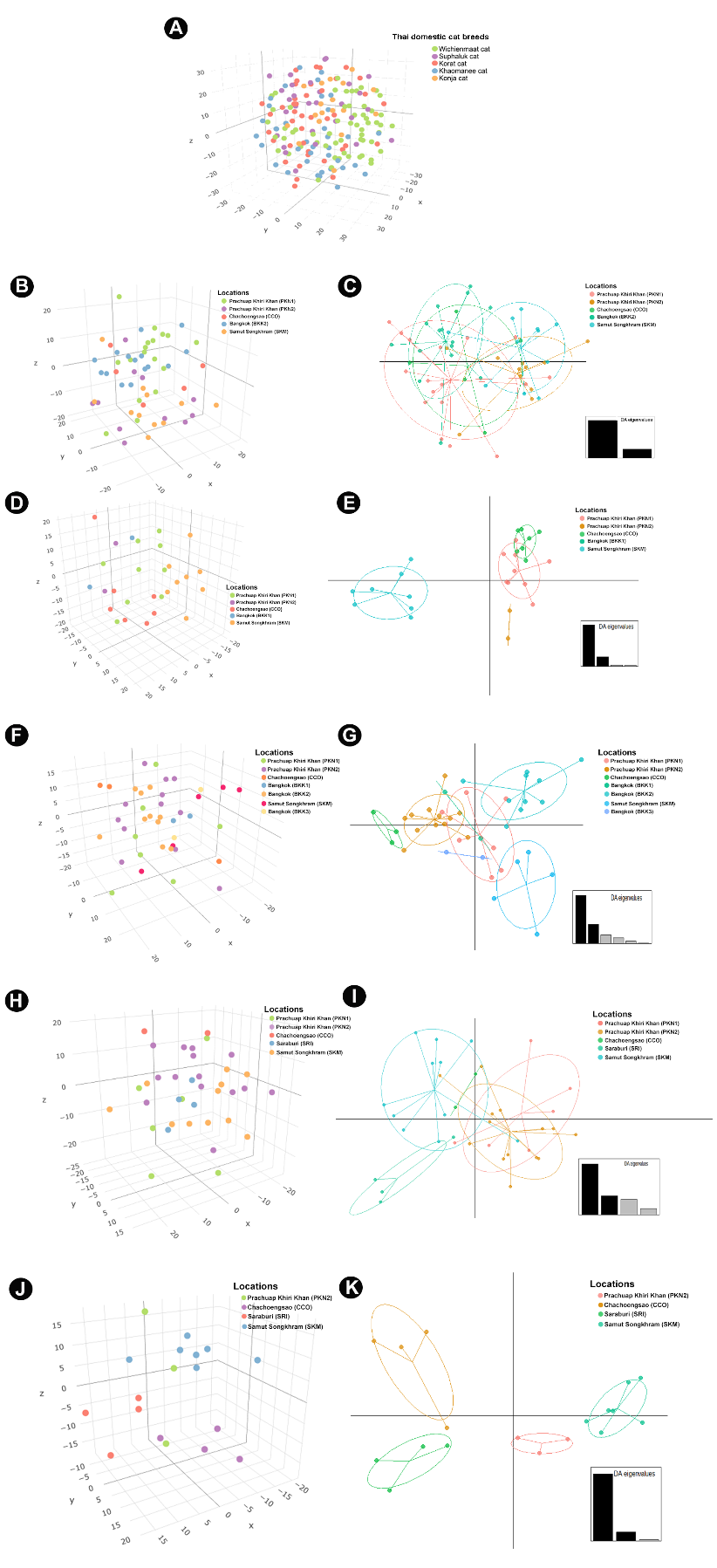


**Supplementary Fig. 4** Genetic structures of five Thai domestic cat breeds separated by breed and location revealed by (A, B, D, F, H and J) principal component analysis (PCoA) and (C, G, E, I, and K) the discriminant analysis of principal components (DAPC).


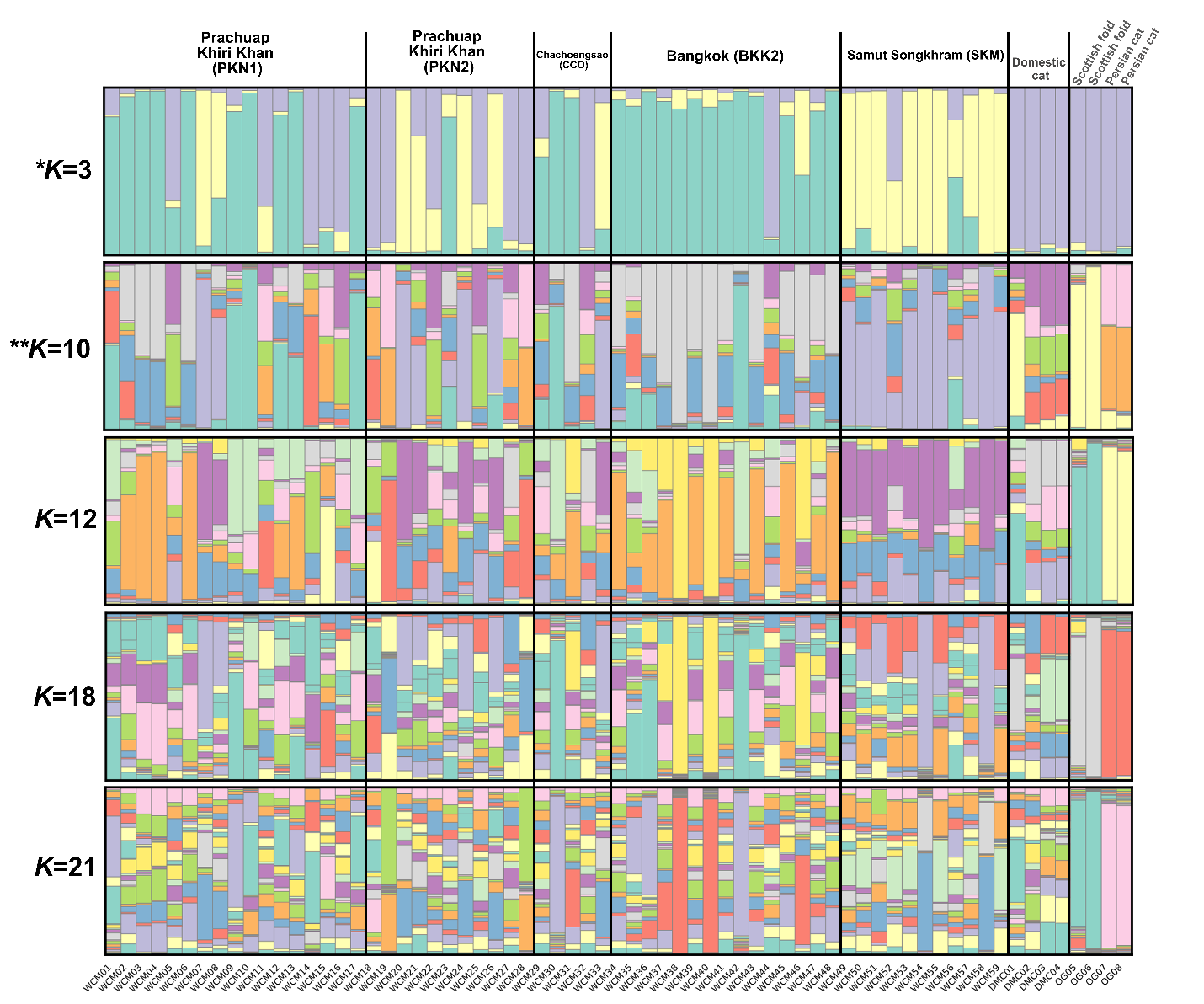


**Supplementary Fig. 5** Population structure of Wichienmaat cat breeds separated by location. The best plot from Evanno’s Δ*K* (*) and ln P(*K*) (**).


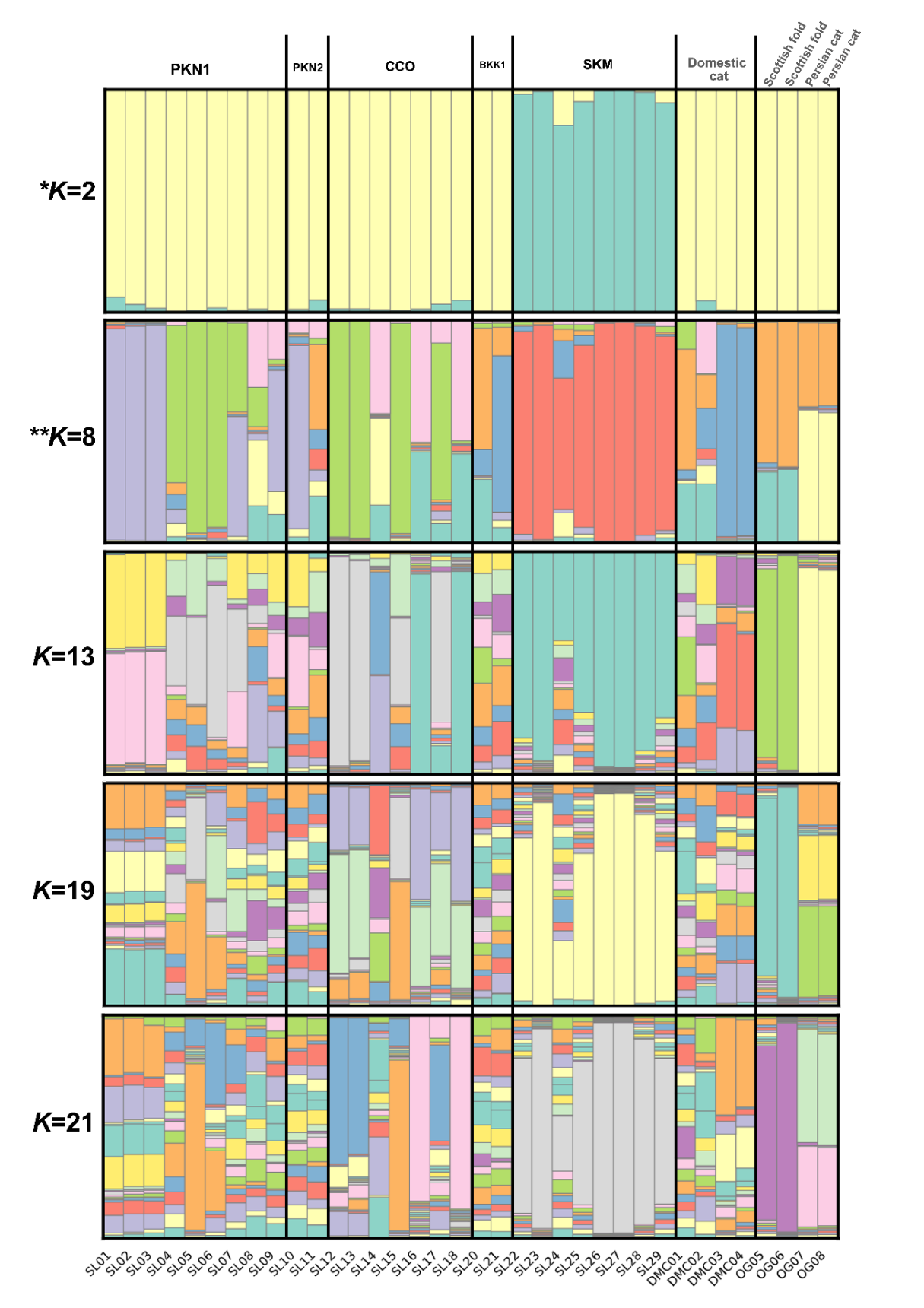


**Supplementary Fig. 6** Population structure of Suphaluk cat breeds separated by location. The best plot from Evanno’s Δ*K* (*) and ln P(*K*) (**).


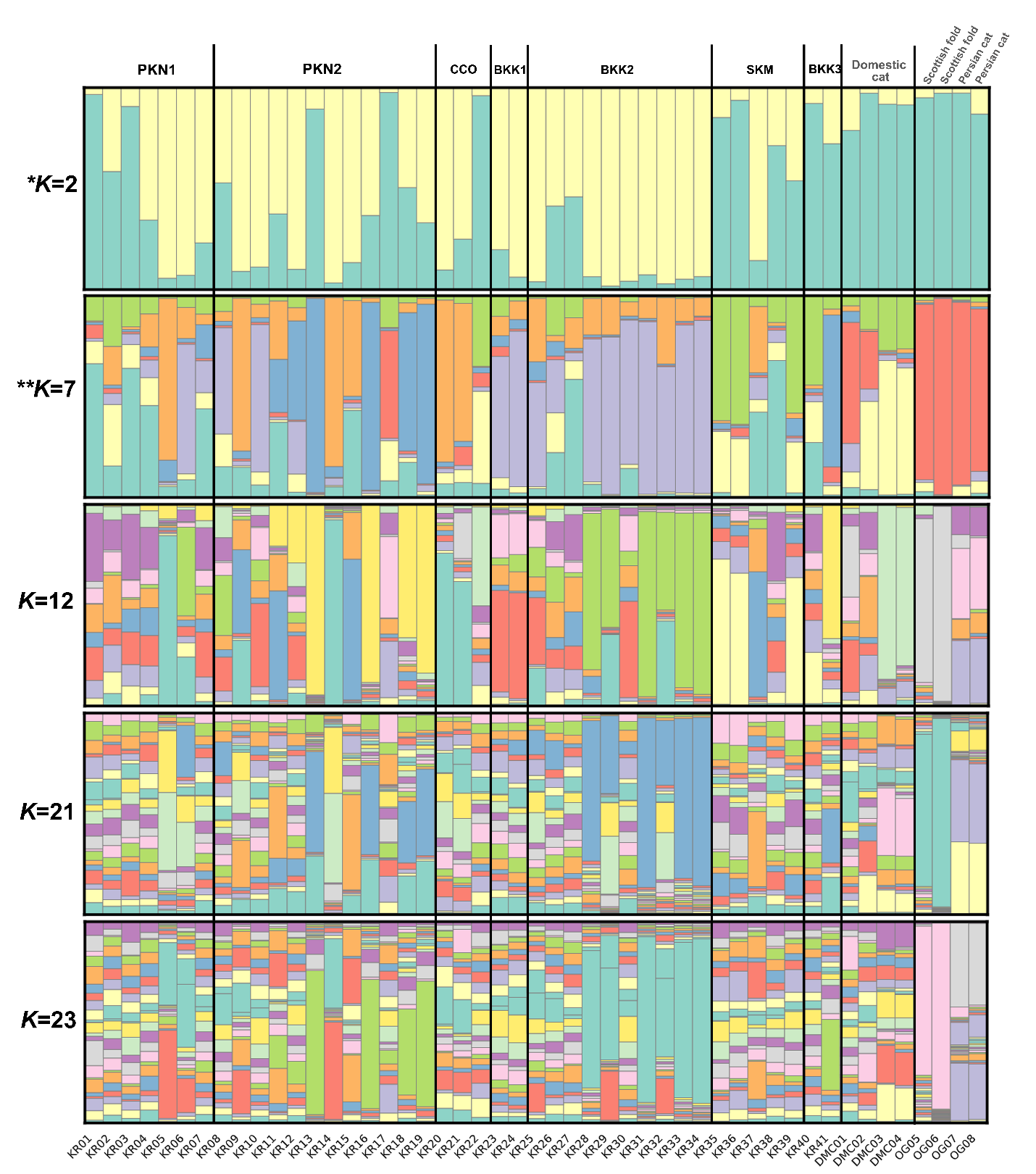


**Supplementary Fig. 7** Population structure of Korat cat breeds separated by location. The best plot from Evanno’s Δ*K* (*) and ln P(*K*) (**).

**
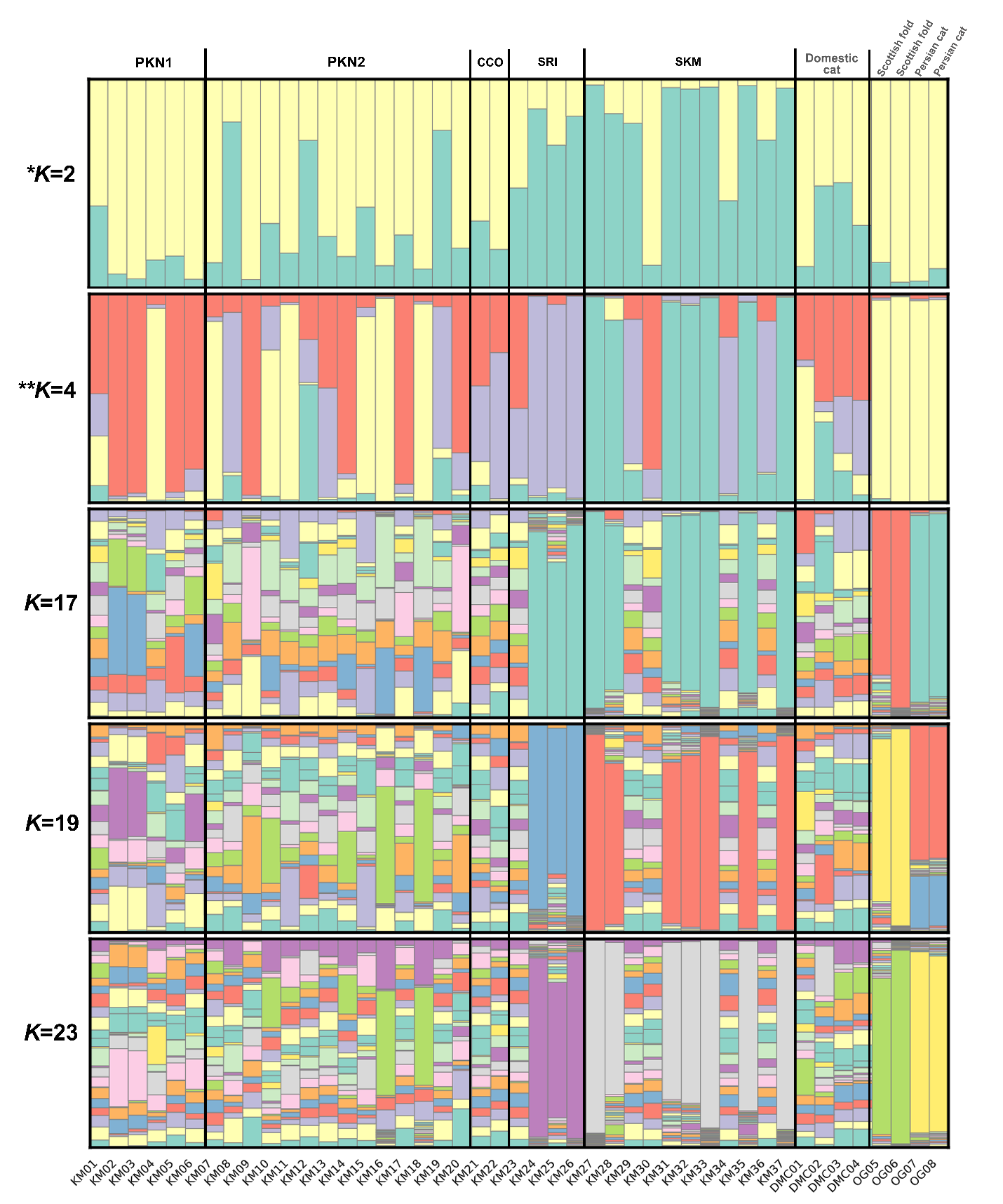
**

**Supplementary Fig. 8** Population structure of Khao-manee cat breeds separated by location. The best plot from Evanno’s Δ*K* (*) and ln P(*K*) (**).

**
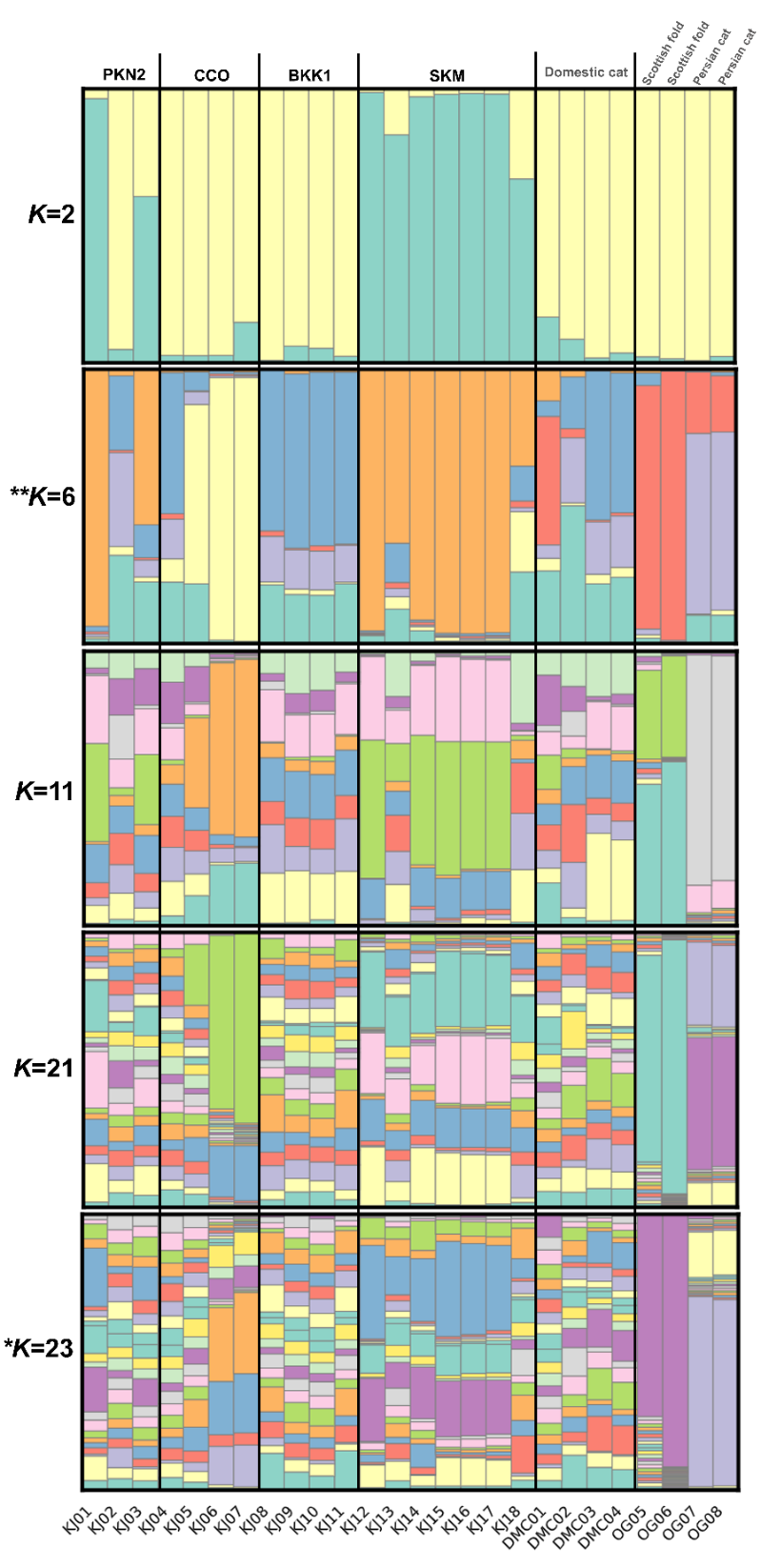
**

**Supplementary Fig. 9** Population structure of Konja cat breeds separated by location. The best plot from Evanno’s Δ*K* (*) and ln P(*K*) (**).


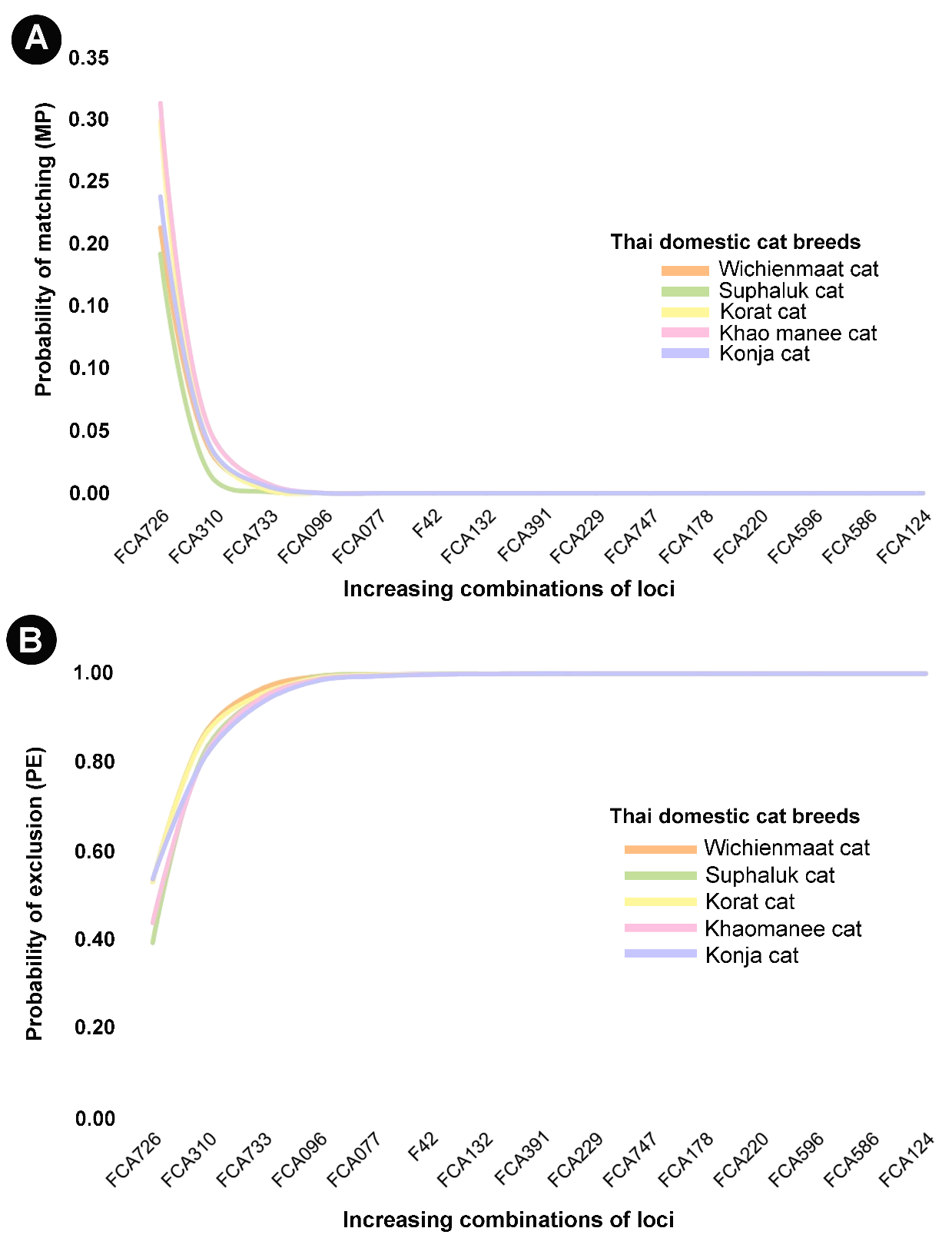


**Supplementary Fig. 10** (A) Matching probability (MP), and (B) probability of exclusion (PE) values of 15 microsatellite loci, estimated using GenAIEx version 6.5 [1] software.

1. Peakall R, Smouse PE. GenAlEx 6.5: genetic analysis in Excel. Population genetic software

for teaching and research–an update. Bioinformatics. 2012;28:2537–2539.


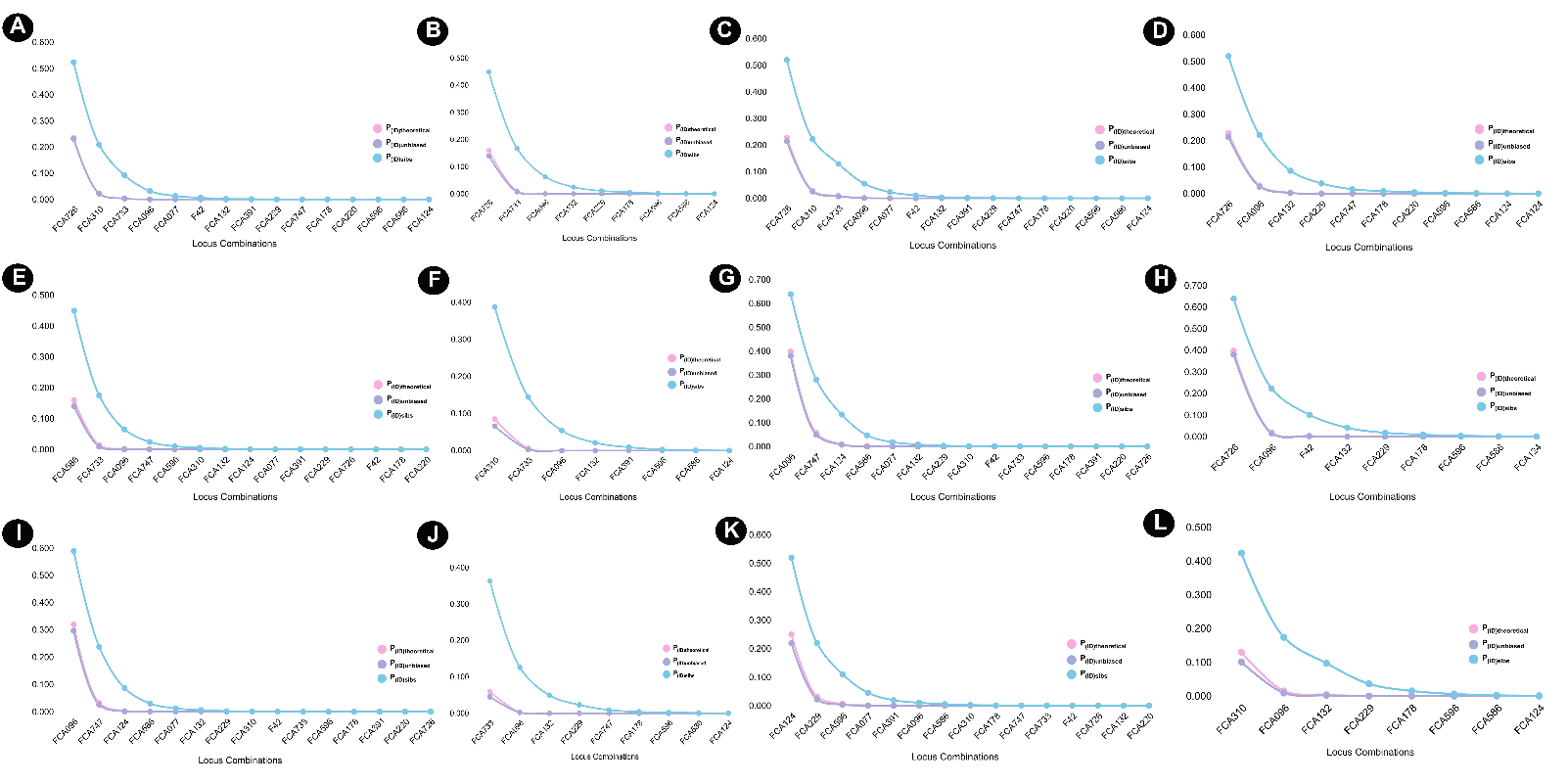


**Supplementary Fig. 11** The theoretical probability of identity (P_(ID)theoretical_), unbiased probability of identity (P_(ID)unbiased_), and probability of identity between siblings (P_(ID)sibs_) based on 15 microsatellite loci (A, C, E, G, I, and K) and P_(ID)theoretical_, P_(ID)unbiased_, and P_(ID)sibs_ based on microsatellite loci after decreased (B, D, F, H, J, and L) of 184 Thai domestic cats (*Felis catus*) and each Thai domestic cat breed calculated using GenAlEx version 6.5 [1] and GIMLET version 1.3.2 [2] software.

1. Peakall R, Smouse PE. GenAlEx 6.5: genetic analysis in Excel. Population genetic software

for teaching and research–an update. Bioinformatics. 2012;28:2537–2539.

2. Valière, N. gimlet: a computer program for analysing genetic individual identification data.

Mol Ecol Notes. 2002;2:377–379.


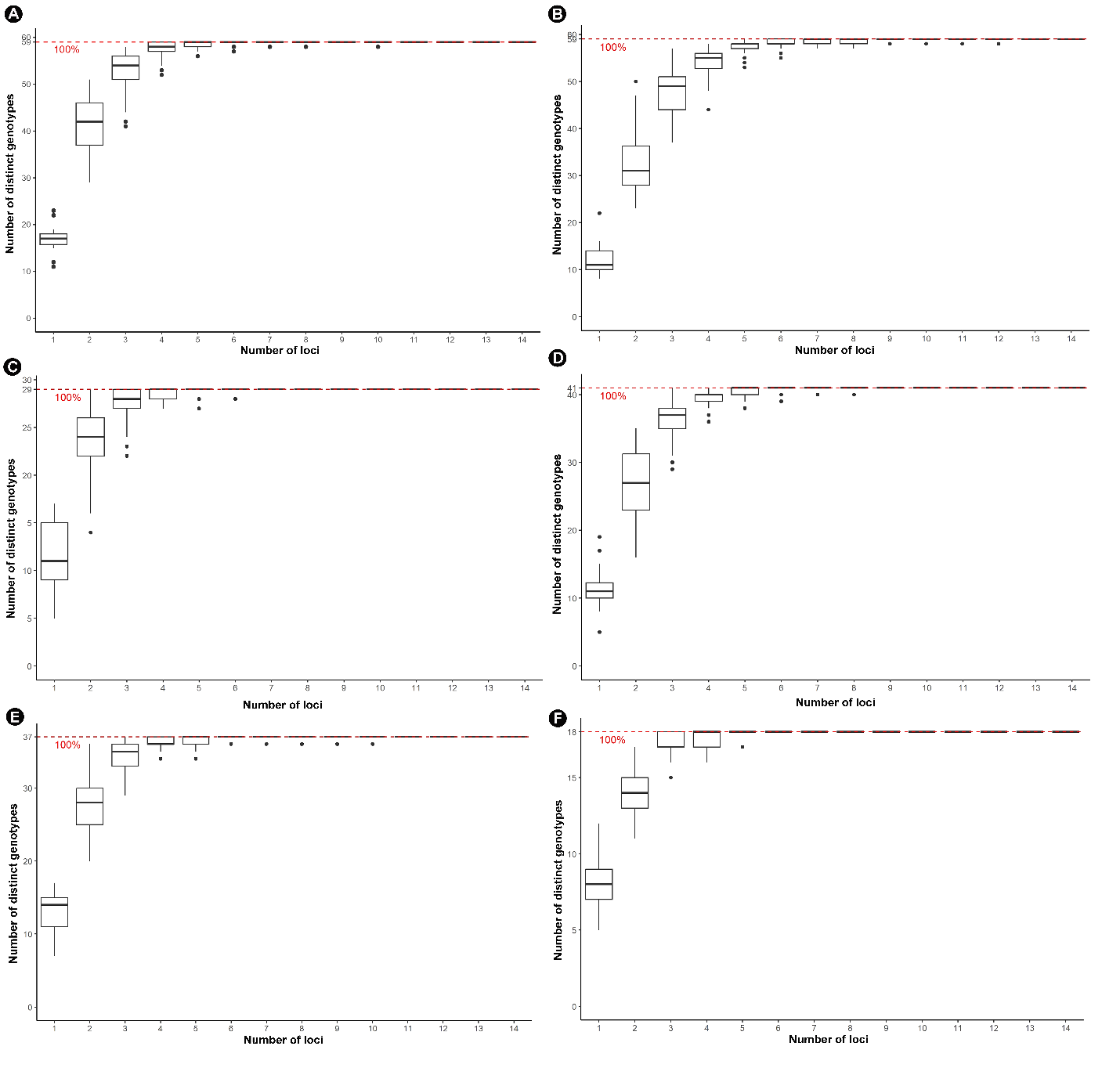


**Supplementary Fig. 12** Genotype accumulation curve to simulate the effects of locus drop out on genotyping. (A) All five breeds, (B) Wichienmaat cat, (C) Suphaluk cat, (D) Korat cat, (E) Khao-manee cat, and (F) Konja cat.


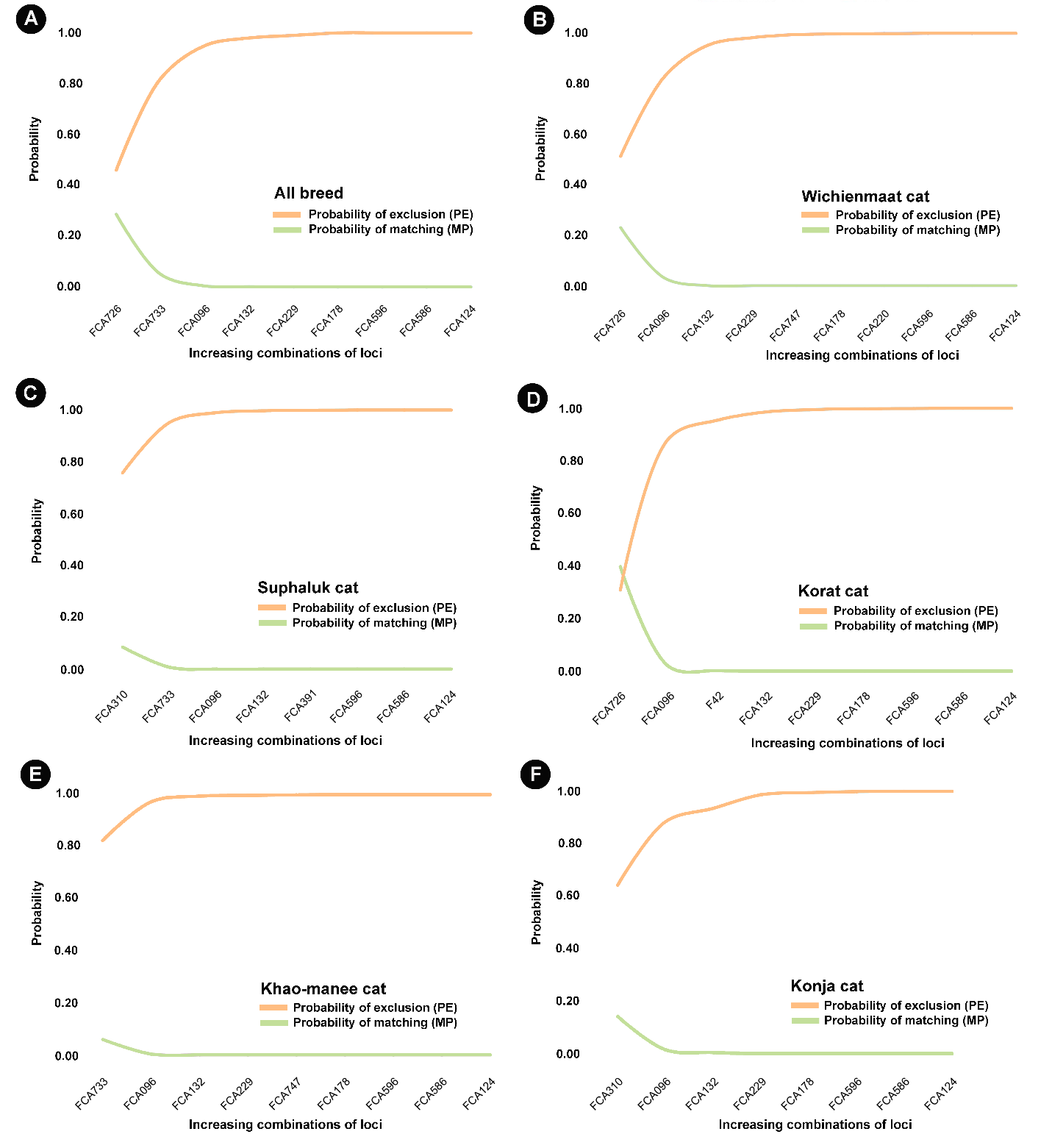


**Supplementary Fig. 13** MP (green line) and PE (orange line) values of decreased microsatellite loci, estimated using GenAIEx version 6.5 [1] software (A, B, C, D, E, and F).

1. Peakall R, Smouse PE. GenAlEx 6.5: genetic analysis in Excel. Population genetic software

for teaching and research–an update. Bioinformatics. 2012;28:2537–2539.


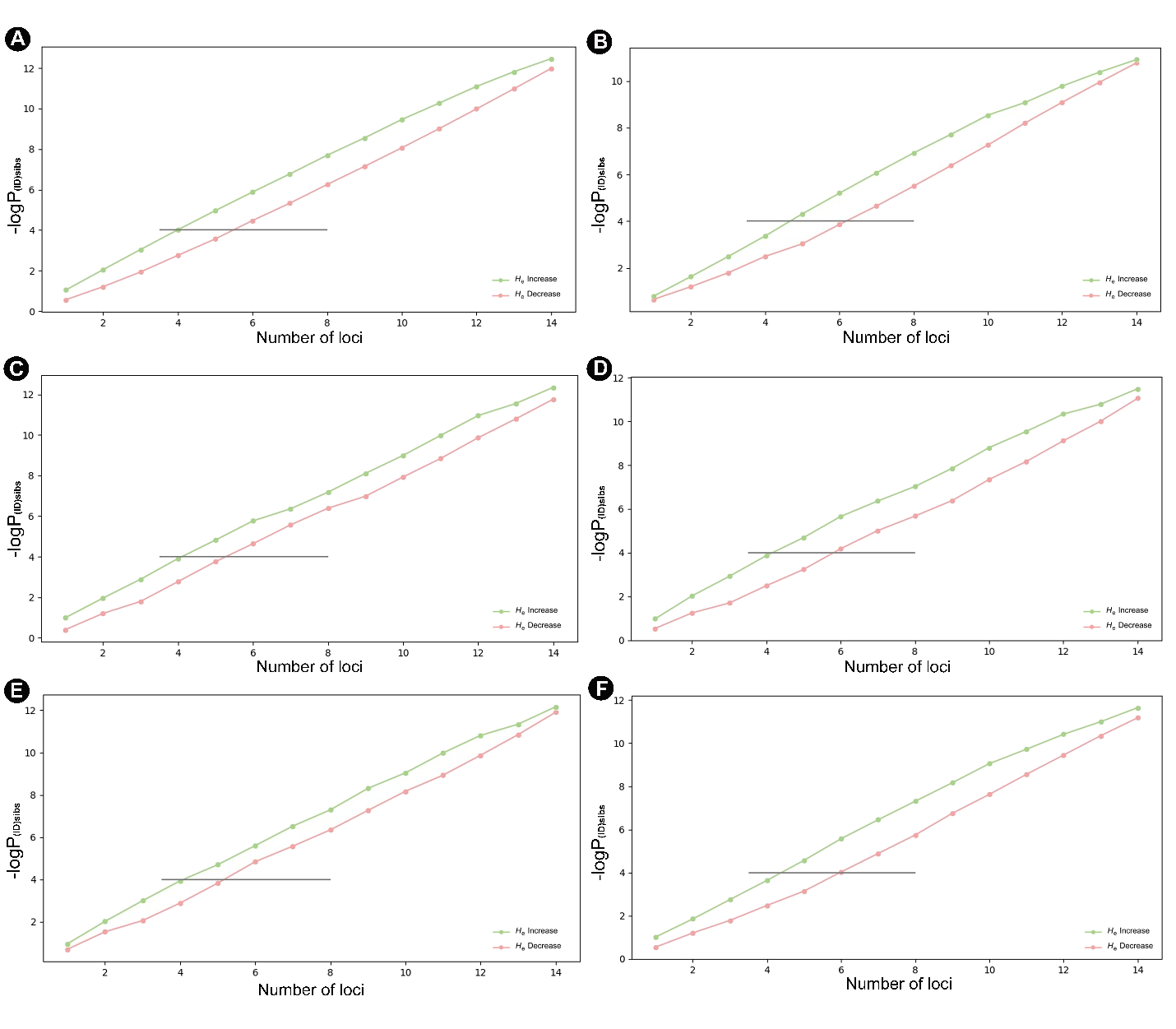


**Supplementary Fig. 14** Probability of identity between siblings (P_(ID)sibs_) value computed for sets of up to 15 loci, beginning with the marker with the lowest *H*_e_ value (pink) and ending with that with the highest *H*_e_ value (green). (A) All five breeds, (B) Wichienmaat cat, (C) Suphaluk cat, (D) Korat cat, (E) Khao-manee cat, and (F) Konja cat.


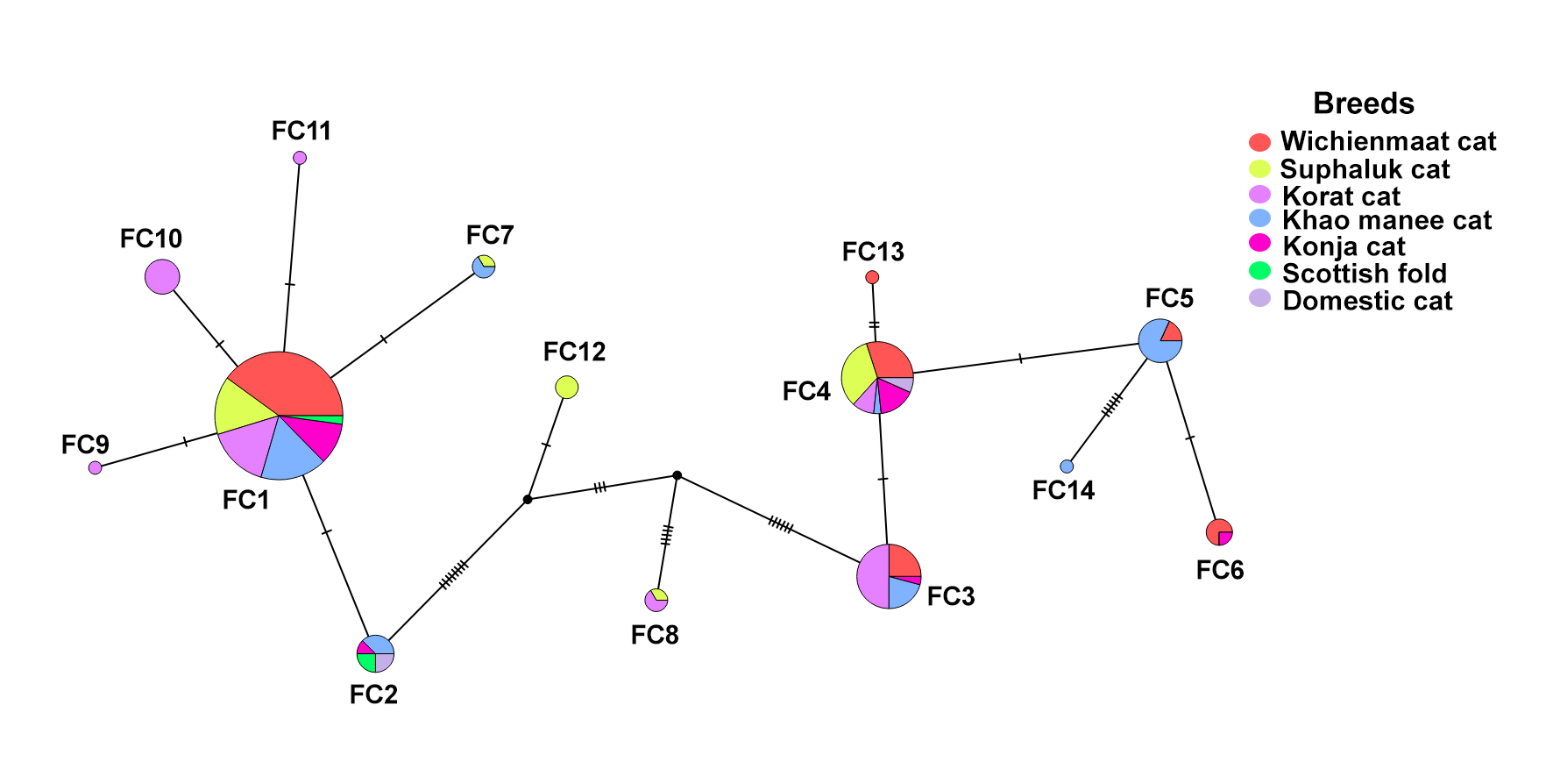


**Supplementary Fig. 15** Haplotype network of five Thai domestic cat breeds based on mitochondrial DNA D-loop sequences.


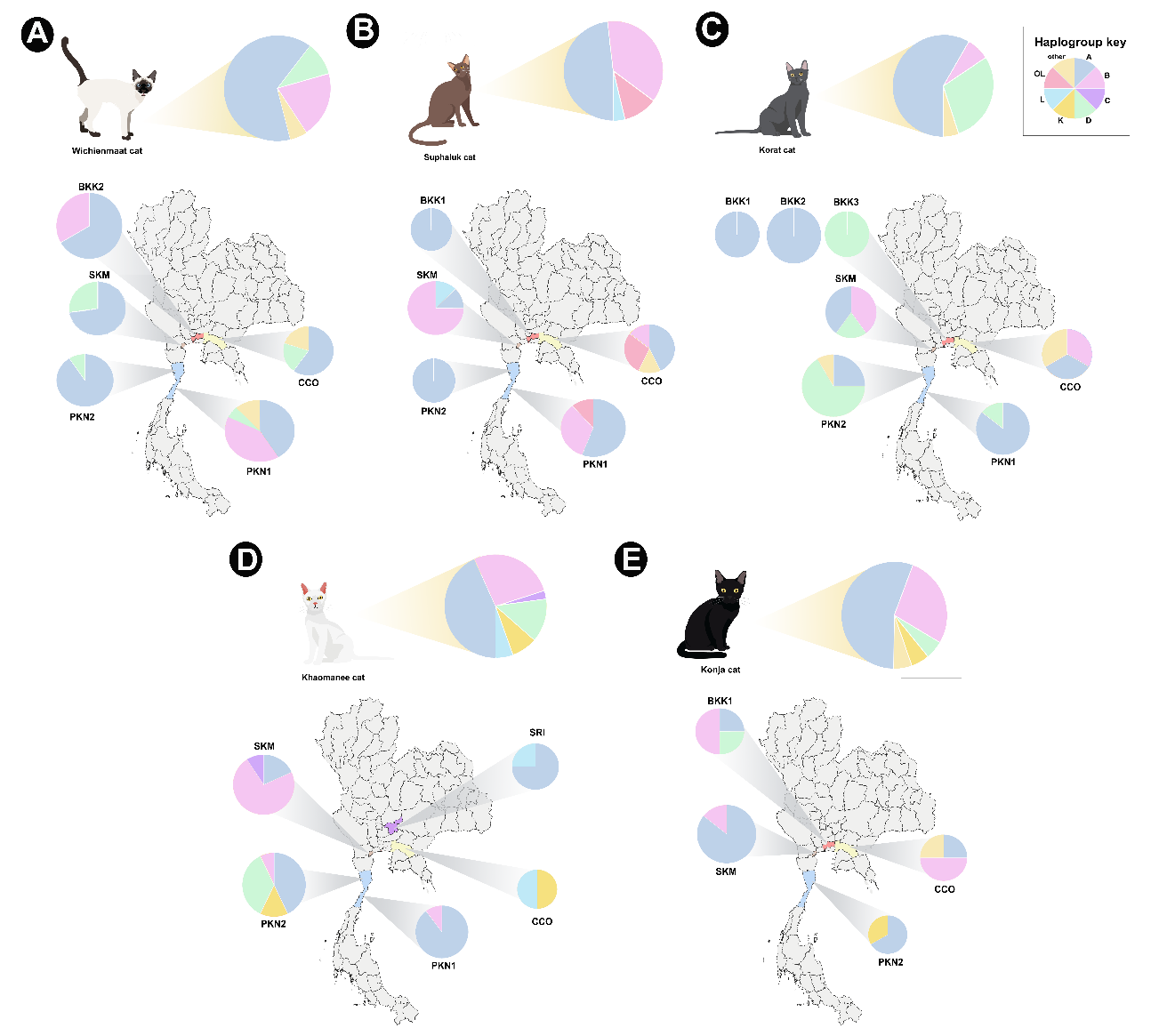


**Supplementary Fig. 16** Haplogroup pattern based on mitochondrial DNA D-loop sequences of five Thai domestic cat breeds. five locations of Wichienmaat cat (A), five locations of Suphaluk cat (B), seven locations of Korat cat (C), five locations of Khao-manee cat (D), and four locations of Konja cat (E).

**
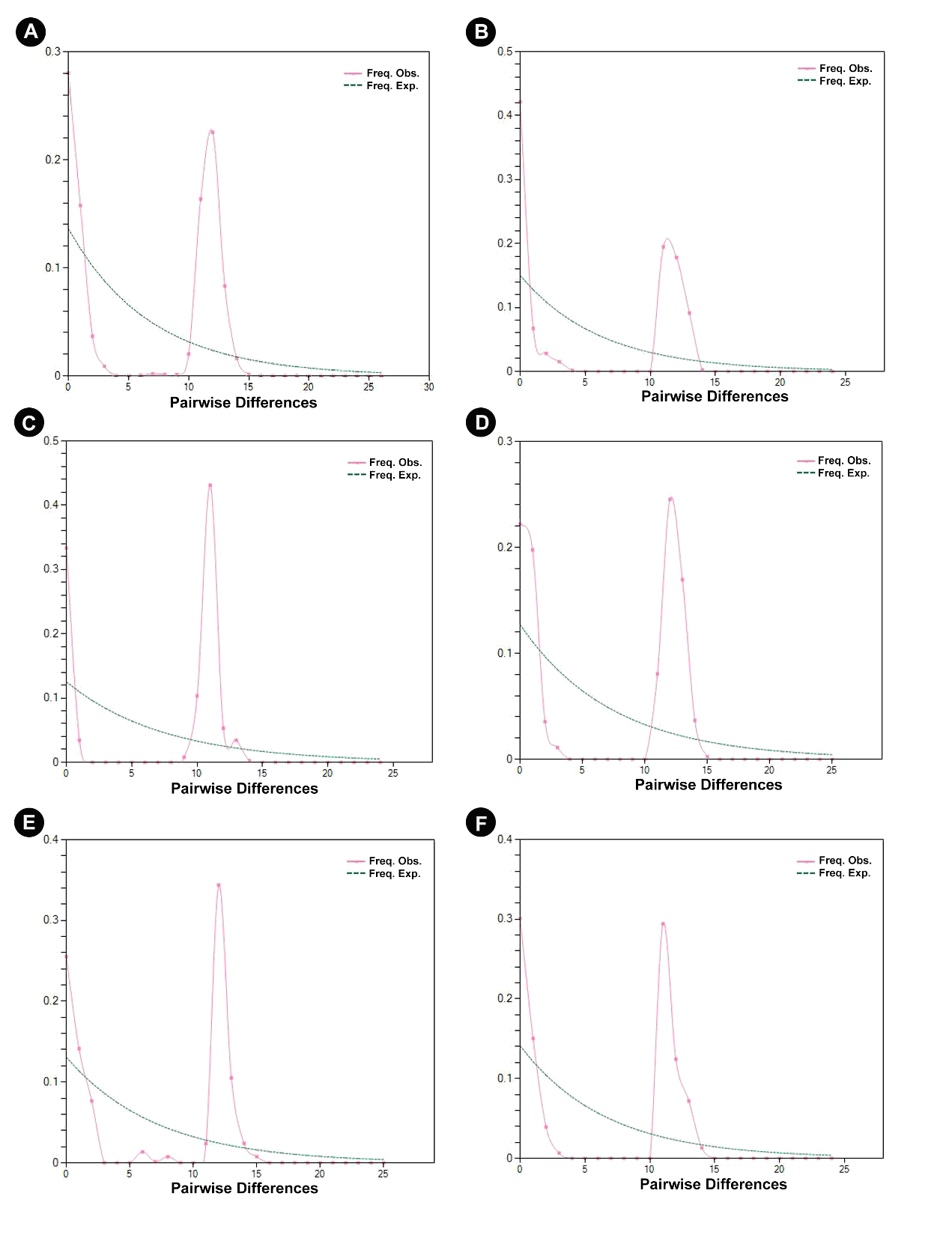
**

**Supplementary Fig. 17** Mismatch distribution of the mitochondrial DNA D-loop sequences in 6 datasets of the Thai domestic cat populations: (A) all populations, (B) Wicheinmaat, (C) Suphaluk, (D) Korat, (E) Khao-manee (F) Konja datasets. The *x*-axis represents the number of pairwise differences (mismatches), and the *y*-axis represents the frequency of these differences. The distribution of frequencies of observed mismatches (pink line) is compared to those of frequencies of expected mismatches (green line).

_
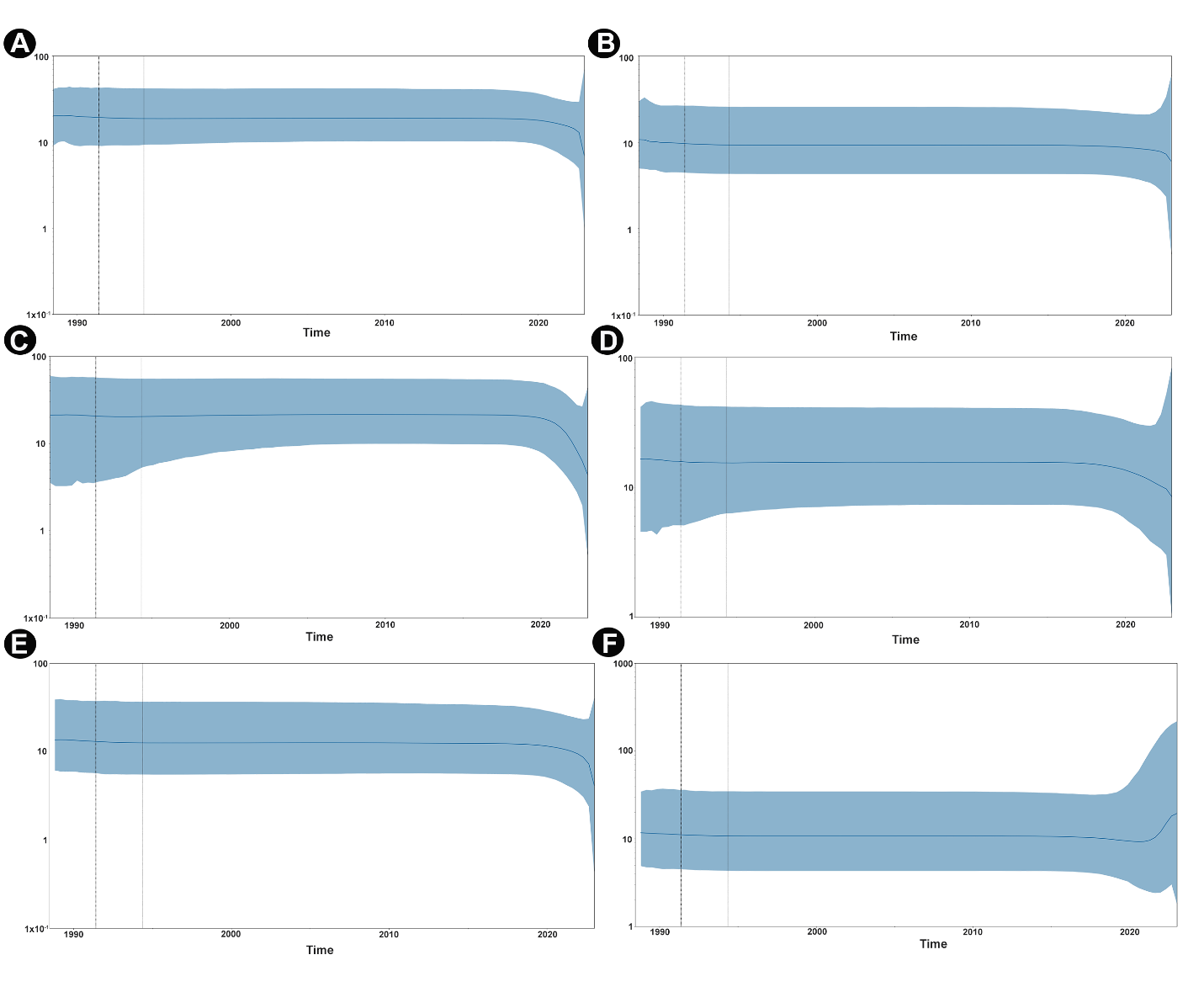
_

**Supplementary Fig. 18** The historical demographic fluctuations of the mitochondrial DNA D‐loop sequences of Thai domestic cat breeds determined using Coalescent Bayesian Skyline analysis. The median effective breeds size is delimited by the black lines. The blue shaded area delimits the upper and lower bounds of the 95% highest posterior density interval. The *x*-axis represents time in years and the *y*-axis is displayed in logarithmic scale.
